# Brain synchronization during perception of facial emotional expressions with natural and unnatural dynamics

**DOI:** 10.1371/journal.pone.0181225

**Published:** 2017-07-19

**Authors:** Dionysios Perdikis, Jakob Volhard, Viktor Müller, Kathrin Kaulard, Timothy R. Brick, Christian Wallraven, Ulman Lindenberger

**Affiliations:** 1 Center for Lifespan Psychology, Max Planck Institute for Human Development, Berlin, Germany; 2 Department of Human Perception, Cognition and Action, Max Planck Institute for Biological Cybernetics, Tübingen, Germany; 3 Department of Brain and Cognitive Engineering, Korea University, Seoul, Republic of Korea; Universitatsklinikum Tubingen, GERMANY

## Abstract

Research on the perception of facial emotional expressions (FEEs) often uses static images that do not capture the dynamic character of social coordination in natural settings. Recent behavioral and neuroimaging studies suggest that dynamic FEEs (videos or morphs) enhance emotion perception. To identify mechanisms associated with the perception of FEEs with natural dynamics, the present EEG (Electroencephalography)study compared (i) ecologically valid stimuli of angry and happy FEEs with natural dynamics to (ii) FEEs with unnatural dynamics, and to (iii) static FEEs. FEEs with unnatural dynamics showed faces moving in a biologically possible but unpredictable and atypical manner, generally resulting in ambivalent emotional content. Participants were asked to explicitly recognize FEEs. Using whole power (*WP*) and phase synchrony (Phase Locking Index, *PLI*), we found that brain responses discriminated between natural and unnatural FEEs (both static and dynamic). Differences were primarily observed in the timing and brain topographies of delta and theta PLI and WP, and in alpha and beta WP. Our results support the view that biologically plausible, albeit atypical, FEEs are processed by the brain by different mechanisms than natural FEEs. We conclude that natural movement dynamics are essential for the perception of FEEs and the associated brain processes.

## Introduction

Human social behavior can be viewed as an ongoing interaction among individuals through a flow of coordinated processes of action and perception such as speech, gestures and facial expressions, which serve to communicate our intentions, emotions, and experiences [1]. Brain processes have evolved to facilitate social coordination [1–4]. Here, we focus on one class of such processes, namely, the perception of facial emotional expressions (FEEs), and study to what degree the associated brain mechanisms are specific to the natural dynamics of FEEs, with implications for related experimental designs in cognitive, affective and social neuroscience.

Most experimental studies on FEEs’ perception have relied on static stimuli, that is, still images, such as those developed by Ekman and Friesen (1976) [5] (see [6–10] for comprehensive reviews). Yet recent behavioral and neuroimaging studies suggest that dynamics may be critically important for the perception of FEEs (see [11] for a review). Behavioral and brain responses to dynamic stimuli, that is, videos or artificial morphs, seem to be stronger than brain responses to static stimuli. Behaviorally, dynamic stimuli lead to stronger spontaneous mimicry [12], higher arousal ratings [13], and enhanced emotion perception [14–16] compared to static stimuli. At the neural level, several fMRI studies [17–20] revealed that dynamic expressions consistently result in higher activity than static expressions in several brain areas. A meta-analysis [21] localized these differences in the FFG, middle temporal gyri, STS and amygdala. These enhanced BOLD activations have been attributed to larger amounts of information conveyed by fluid facial motion [18,19], as well as increased attentional demands [20] for dynamic stimuli. The latter explanation is in line with an ERP (Event Related Potentials) study [22] that found an amplitude increase in early posterior negativity (EPN, 200–300ms post stimulus) and in late positive complex (LPC, 350–600 ms) in response to dynamic expressions, concluding that additional attentional resources are recruited for processing of dynamic FEEs. Similarly, an ERP study with fMRI-constrained regional source analysis [23] found that the Late Posterior Positivity “was more widespread and temporally prolonged for dynamic compared to static faces of disgust and happiness”. In summary, several studies have shown differences of “quantity” in the perception of FEEs and its brain correlates (e.g., enhanced emotion perception and mimicry, arousal ratings, increased attentional demands, higher brain activity or more widespread and temporally prolonged activations due to larger amounts of information and motion).

From a different perspective, converging evidence from a study on a brain lesion patient [24], a PET study [25], a Macaque fMRI study [26], a Transcranial Magnetic Stimulation (TMS) study [27] and a Steady State Visually Evoked Potential (SSVEP) study [28] suggests that perception of static and dynamic FEEs does not differ only in a quantitative manner, but also relies on qualitatively different processes. In particular, the recognition of dynamic FEEs is thought to involve the perception of overt motion, whereas static FEE processing may involve motor imagery to compensate for the missing motion. Moreover, the SSVEP study [28] also found that static FEEs are processed at earlier latencies than dynamic FEEs, i.e. the associated processes differ in their timing as well. Finally, PET [25] and Macaque fMRI [26] studies showed that different networks are activated during the perception of static or dynamic FEEs, suggesting that these networks correspond to distinct mental strategies.

All of the studies mentioned above have contrasted FEEs of either natural or morph dynamics to static images. Yet very few studies have focused on how FEEs’ dynamics specifically influence perception. In [29], participants were shown to be better at identifying conversational expressions from a video of a natural FEE than from reversed-playback or frame-scrambled versions of the same video. Similarly, another study [14] included a condition in which the perception of dynamic FEEs was disturbed by inserting masks between each pair of video frames, which led to lower identification accuracy than in the static and unmasked dynamic conditions. These authors also showed that subtle emotional expressions particularly benefit from a dynamic presentation. In [16] the authors used MEG to compare predictable dynamic, unpredictable (frame-scrambled) dynamic and static stimuli, and found stronger event-related field components in response to predictable dynamic stimuli. Differences were already present in low-level visual areas (primary visual cortex) as early as 165 ms post stimulus, and spread towards higher-level regions associated with face perception (bilateral visual cortex, posterior fusiform gyrus and right posterior STS but also bilateral premotor cortex) within the next 100 ms (peaking at 237 ms post stimulus). The authors of [16] suggest that perception of FEEs relies on brain processes that are sensitive to *coherent*, *predictable movement trajectories*. Following recent findings in an fMRI study [30] and related theoretical predictions [31], they postulate that these movement trajectories are organized in a time hierarchy, by which sensory trajectories stimulating lower-level visual cortex are integrated in higher-level areas at slower timescales.

However, a possible confounding factor of the last three studies could lie in the way the dynamic control stimuli were constructed, both in terms of the coherence and time order of movement trajectories, and in the resulting biological plausibility of the stimuli. In particular, reversed-playback [29] is a very specific movement transformation which preserves coherence (albeit in the reverse time order). Similarly, masking [14] partially disrupts the coherence preserving the time order, and frame scrambling [16,18,29] disrupts both coherence and time order. In all cases except reverse playback, the resulting stimuli lack biological plausibility, whereas both reverse playback and masking fail to impair predictability. The approach taken in our study (see below) loosely resembles the approach taken in [32], which used computationally generated random–but still biologically plausible–FEEs. They show behavioral evidence that “dynamic facial expressions of emotion transmit an evolving hierarchy of “biologically basic to socially specific” information over time”, but do not examine the neural processes at work. To our knowledge, no associated brain data has been published using such a paradigm, leaving open the question of whether previously observed differences in the brain activations could be due to the biological implausibility of the control stimuli employed in the studies of the previous paragraph.

The present EEG study attempts to evaluate whether the brain mechanisms associated with FEEs’ perception are specific to the natural dynamics of FEEs, as these have evolved biologically and socially, while addressing the problem of the possible confounding factors described above. We build on the general idea that disturbing a process helps to understand its dynamics. Thus, in the context of an oddball task that required explicit recognition of FEEs, we compared (i) natural movement dynamics of FEEs presented in video clips of a short duration that are taken from an ecologically validated database [33], to two conditions with impoverished dynamics, namely, (ii) FEEs with unnatural dynamics, and (iii) still images. FEEs with unnatural dynamics depicted biologically possible facial expressions with the same amount of movement as in the original FEEs, in which movement components have been shifted in time to distort natural movement coordination. The resulting stimuli have an ambivalent or “fuzzy” emotional content. Static FEEs were considered as a special, marginal case of stimuli of “unnatural” (i.e., impaired) dynamics. This categorization follows [25], which characterized static FEEs as “degraded social stimuli” compared to their dynamic counterparts. Our general expectation was that both comparisons of FEEs stimuli of natural dynamics with those of impaired dynamics (either static or unnatural dynamics) would reveal that the dynamics of FEEs differentiate the associated brain processes. In particular, we expected that the observed differences in brain responses could not be attributed solely to the existence, absence or amount of stimulus variation over time, nor to the raw quantity of static information.

To test these hypotheses, we used measures of brain synchrony in the time-frequency domain because, unlike ERP, they are able to quantify brain processes on different time scales. Time-frequency techniques therefore can take full advantage of the fine time resolution of EEG, and are sensitive to the stimuli’s dynamics. In particular, we opted for two measures that capture complementary aspects of brain synchrony related to amplitude and phase: Whole Power (*WP*) and Phase Locking Index (*PLI*). *WP* is a measure of the amplitude of brain oscillations due to their local neural synchronization. Relative change in WP of these oscillations in response to a stimulus is related to changes of the parameters of these oscillations [34–36]. *PLI*, in turn, reflects how strictly the timing of brain oscillations is related to the stimulus [36–38] by measuring the consistency of phase across stimulus time-locked trials. EEG studies that analyzed the influence of FEEs on brain oscillations found that perception of emotional compared to neutral expressions led to increased delta and theta power between about 150 and 300 ms post-stimulus [39,40]. Their findings suggest that delta reflects updating of the stimulus, whereas theta responds to the emotional significance of FEEs. However, those studies used static stimuli, and, to our knowledge, there exists no study utilizing time-frequency measures on dynamic FEEs.

In addition, we used a statistical method suitable for exploratory studies involving multivariate data, namely “mean-centering task Partial Least Squares (PLS)” [41,42]. We anticipated that in the comparison of natural dynamic versus static FEEs, we would find differences mainly in the timing of the perceptual process, in line with the previous findings of [28], as static FEEs depict the “full-blown” expression at the very first video frame, whereas the natural dynamic FEEs develop gradually over time. We therefore expected stimulus-driven activity and task-relevant activations, as captured by *PLI* and *WP* respectively, to be higher for static FEEs at early latencies, but more prolonged towards later latencies for natural dynamic FEEs. In the comparison of natural versus unnatural dynamic FEEs, we anticipated evidence for a prolonged and perhaps strenuous effort of the brain to sample the incoming unfamiliar dynamics of unnatural FEEs and link them to previous experiences of facial emotional expressions, in order to make sense of them.

## Methods

### Participants

We tested twenty-nine female right-handed participants with normal or corrected-to-normal vision and without any history of neurological or psychiatric illness or substance abuse. We decided to include only women in the study, because they are known to react more strongly to facial expressions than men [43]. The objective was to eliminate gender differences in expression perception as a variability factor outside the scope of this study, at the admitted expense of generalizability to the male population. Four participants were excluded because of an insufficient number of usable trials (see below). The remaining twenty-five participants had a mean age of 25.3 years (SD = 3.3 years, range from 20 to 34 years). The Ethics Committee of Max Planck Institute for Human Development approved the study, which was performed in accordance with the ethical standards laid down in the 1964 Declaration of Helsinki. All participants volunteered for this experiment and gave their written informed consent prior to their inclusion in the study. Participants were paid eight Euros per hour.

### Stimuli generation

We selected videos with four different actors (two male, two female) from the MPI Facial Expression Database [33], which has been constructed by means of an acting protocol that aims to make the resulting expressions as natural as possible. For each actor, we used videos of two expressions, angry and happy, and created stimuli of three motion types: natural dynamic, unnatural dynamic and static. The natural dynamic stimuli show a natural transition from neutral to emotional expression. The unnatural dynamic stimuli were created with the Face Modeling GUI software (Version 0.2r26 [44]), which, in turn, relies on the Deformable Model Library [45]. In order to create those stimuli we first created an active appearance model of each actor [46,47]. Such a model describes the video of a face as a set of orthogonal components, which together explain 99% of the covariance in a) the shape of the face, and b) the color of all pixels on the face (across all frames of the video). The models for the videos of the 4 actors we used consisted of 8, 13, 12 and 14 components, respectively. With the help of these models, we tracked the movements of each expression, which results in a set of simultaneous trajectories of each component. That is, each frame of the original video is described by a set of coefficients that each capture the activation of one component in that frame. To create stimuli with unnatural dynamics, we independently manipulated the trajectories of these components, disturbing the coherence among them. We achieved this by shifting each trajectory within a video cyclically in time for a random number of time points and by inverting it with a probability of 0.5. The resulting trajectories have the same “amount of movement” in the sense that their power spectra are almost identical to the ones of the original trajectories. To avoid jerky movements, the beginning and end of each component were blended together. To avoid differences between the motion types, we used the same software to create the stimuli for natural dynamic stimuli as for the unnatural dynamic ones, but without manipulating the movement trajectories. For static stimuli, we repeated the last frame of the respective natural dynamic expression. All stimuli had duration of 1240ms (31 frames, 40 frames per second, mpeg-4), as a compromise, which allowed all natural dynamic stimuli to follow approximately a trajectory from the neutral position to an expressional peak. Characteristic stimuli are included in S1–S3 Videos).

### Procedure

Participants were seated comfortably on a chair at a distance of 55cm from a 17-inch computer screen, on which the expression stimuli were displayed. First, participants went through a stimuli validation session, where they were asked to rate the stimuli on a scale from 1 to 7 by answering six questions (see Table 1). Two questions evaluated the emotional content of the stimuli, two the total amount of movement observed or implied (for the static FEEs) in the stimuli (one each for movements of the eyes and mouth), and the last two the “naturalness” of the expressions displayed in the stimuli. Participants viewed the stimuli as many times as they wanted (thus getting accustomed to the stimuli before the EEG session), in a random order, and responded to the six questions in a random order as well. This validation session was followed (after fitting the EEG cap to the participants) by the main EEG session, which consisted of an oddball task with explicit emotional expression recognition. Participants viewed the stimuli for a duration of 1240 ms, followed by a fixation cross with a variable duration of uniform distribution between 1200 and 1800 ms. One class of emotional expression (the ‘standard expression’) was shown more frequently (ratio 10:3) than the other one (the ‘deviant expression’). Participants were asked to respond with a button press of the right hand to the deviant stimuli. They were instructed to do so as fast as possible, but only if they were sure that they are correct, introducing, thus, a bias towards misses. We registered the errors (false alarms and misses) and the reaction times. The task was organized in six blocks. In half the blocks the deviant expression was anger, in the other half it was happiness, unlike motion conditions that were mixed within blocks. The participants were informed before each block to which expression they should respond. In every block, each deviant stimulus was presented 3 times and each standard stimulus 10 times, resulting in 152 trials per block, while there were 30 standard and 10 deviant trials for each stimulus in total. The ordering of the blocks and the stimuli within each block was pseudo-random, with the restriction of no more than two consecutive blocks of the same deviant expression.

**Table 1:** Questions for stimuli’s validation in English and German, and the corresponding variables.

Variable				
		Questions		
„ Happy “	1		How happy is the person?	Wie frühlich ist die Person?
„ Angry “	2		How angry is the person?	Wie wütend ist die Person?
„Movement“	3		How much is the person moving its eye region?	Wie stark bewegt die Person ihre Augenregion?
	4		How much is the person moving its mouth region?	Wie stark bewegt die Person ihre Mundregion?
„Naturalness“	5		How much sense does the expression of this person make to you?	Wie viel Sinn macht der Gesichtsausdruck der Person?
	6		How natural is the expression of this person?	Wie natürlich ist der Gesichtsausdruck der Person?

### Physiological recording and analysis

Electroencephalographic (EEG) recording took place in an electrically and acoustically shielded cabin. An active 60-channel electrode system was applied to the participants according to the international 10–10 system, with the AFz electrode as ground and right mastoid as reference electrode. To control for blinks and eye movements, vertical and horizontal electrooculograms (EOG) were recorded. All physiological recordings were done with BrainVision Recorder 1.2 (Brain Products, Munich, Germany) at 5000 Hz sampling rate with a band-pass filter ranging from 0.01 to 1000 Hz.

#### Preprocessing

All preprocessing steps were performed with the software BrainVision Analyzer 2.0 (Brain Products, Munich, Germany). EEG data was re-referenced to the average of left and right mastoids, band-pass filtered (0.5 to 150 Hz, 12 dB/oct) and downsampled to 250 Hz. Next, the data was corrected for eye movement and blink artifacts, using the ocular correction independent component analysis (FASTICA algorithm [48]) that is included in the software [49,50]. Subsequently, the data was manually inspected and remaining artifacts were rejected. The data was segmented into epochs ranging from 500 ms pre-stimulus to 1750 ms post-stimulus, leading to epochs of time length *T* = 2.250 sec. Although the task had an “oddball” design, we analyzed only data from standard trials, since we were interested in the brain processes associated with FEEs perception and not in the responses to deviant stimuli. Thus, epochs that belonged to deviant trials or had a false positive response, i.e. all trials that included a motor response, were rejected. This cleared the data from movement-related brain activity. Participants that had less than 20 acceptable trials for any stimulus were excluded from further analysis–this was true for 4 participants out of the 29 tested. Overall, 28.83 trials per participant and stimulus pair were used on average, with a standard deviation of 1.73. Finally, EEG data was standardized within each participant, in order to remove inter-individual differences.

#### Whole power and phase locking index

EEG time series were transformed after pre-processing into complex value time-frequency space with the following complex Gabor window function:

$$G(t)=e^{-\frac{t^{2}}{2\sigma_{t}^{2}}}e^{-j2\pi t},$$

where $\sigma_{t}=1/\left(2\pi \sqrt{2}\right)$ = 0.1125 sec, leading to a time wave length of *λ*_*t*_ = 2*πσ*_*t*_ = 0.7071 sec and a spectral bandwidth of b_f_ = 1/*πσ*_*t*_ = 2.8284 Hz for all frequency bins. We used a frequency vector of values between approximately 0.89 and 20 Hz with a step of Δ*f* = 0.4444 Hz (resulting from the time length of the epochs, since Δ*f* = 1/*T* is the Rayleigh frequency limit [51]). From the resulting complex value signal, the two measures, whole power and phase locking index, were calculated. Whole power (*WP*) is the average power of the signal across all trials:

$$WP(t,f)=\langle {\left|y_{k}(t,f)\right|}^{2}\rangle ,$$

whereby *y*_*k*_(*t*,*f*) is the complex value of trial *k* at frequency *f* and time *t*, and 〈∙〉 indicates averaging across trials. For further analysis, *WP* values were transformed to their natural logarithm. Phase Locking Index (*PLI*) reflects the constancy of phase *φ*_*k*_(*t*,*f*) across trials, independent of power information:

$$PLI(t,f)=|\langle e^{j∙\varphi_{k}(t,f)}\rangle |,\;j=\sqrt{-1}.$$

*PLI* ranges from 0 to 1, where 1 means total phase constancy across trials and 0 means no phase constancy. For further analysis, *PLI* values were logit-transformed. *PLI* was calculated separately for each stimulus before averaging across different stimuli of the same condition. High values of the outcome therefore represent time-frequency points that are constant in phase across trials. Both *WP* and *PLI* values were baseline corrected after transformation by subtracting the mean value of the baseline interval 200 ms prior to stimulus’ onset from each data point (frequency- and channel-wise). The time interval 0–1500 ms post-stimulus was used for further analyses and we selected four frequencies each for the delta (0.9, 1.8, 2.7 and 3.6 Hz; Δf = 0.89 Hz), theta (4.4, 5.3, 6.2 and 7.1 Hz; Δf = 0.89 Hz), alpha (8.4, 9.8, 11.1 and 12.4 Hz; Δf = 1.33 Hz), and beta (14.2, 16.0, 17.8 and 19.5 Hz; Δf = 1.7 Hz) bands, in order to have an equal number of frequency bins for each frequency band. This was important because we used a multivariate analysis (see below) in which higher frequencies would have been over-represented otherwise. Time resolution was also reduced to 40 ms by selecting one out of ten time bins in order to reduce the computational requirements.

#### Partial least squares analysis

We used “*mean-centering task PLS*” (as implemented in MATLAB according to [42]; see also [41]) to analyze the effect of the expression and motion conditions (factors EXPRESSION and MOTION, respectively) on *PLI* and *WP*. In a nutshell, task PLS ("Partial Least Squares") is a multivariate statistical method that is suitable for revealing the relationship between two blocks of data (in our case local *PLI* or *WP* on the one hand and vectors coding for the experimental design on the other hand) and is in increasingly common use in neuroimaging [42]. PLS is particularly suited to (a) highly multivariate data (i.e., with many more variables than observations) (b) that are highly correlated with each other (as is frequently the case for neurological data). PLS uses data-driven resampling-based approaches to determine p-values, and so is robust to violations of the usual distributional assumptions of tools such as MANOVA. Further, PLS generates latent variables for both brain and design (see below and the Results), which permits us in this case to simultaneously model the predictors of affective state, motion dynamics, and interactions between them. Mean-centering task PLS is based on a decomposition of the covariance of the two blocks into a set of new variables that optimally (in a least square sense) relate them, essentially explaining as much of covariance with as few dimensions as possible. The method begins by constructing a brain data matrix. Rows in this data matrix correspond to participants within conditions. Consequently, the data matrix is made up of (*number of participants*) x (*number of conditions*) rows. Each column represents a computed metric for that individual in that condition at a given time point relative to stimulus onset, with one column for each such time point and metric. Each participant’s rows are averaged column-wise within conditions creating a matrix, **M**, with rows corresponding to conditions, and one column for each metric at each time point. A grand average row is removed from all rows of **M**, and the modified matrix **M** undergoes a singular value decomposition U*S*V = SVD(**M**), which yields three matrices: i) the *task design latent variables*, i.e., the orthonormal matrix **V** of the *saliences of the contrasts* describing the relations among the conditions of our design for each contrast, ii) the *brain latent variables*, i.e., an orthonormal matrix **U** of *element saliences* that are proportional to the covariance of each data element with each one of the task contrasts, and iii) the diagonal matrix **S** of *singular values* that are proportional to the variance explained by each contrast. In particular, the ratio of each squared singular value to the sum of squares of all singular values of all LVs describes the percentage of total covariance accounted for by that LV. The number of resulting singular values, one for each contrast, depends on the degrees of freedom of the design. PLS addresses the problem of multiple comparisons for statistical significance by using an omnibus permutation test to determine the number of significant latent variables, and estimates element-wise reliability via bootstrap resampling. A single permutation test is performed to assess the statistical significance of all singular values as a whole–therefore a correction for multiple comparisons is not required, with resampling of the initial data matrices across conditions without replacement. This permutation test yields a *p*-value for each *task latent variable*, i.e., for each contrast. Our two primary hypotheses, each represented by a single PLS analysis, are similarly not subject to multiple testing between the two, because they represent independent *a priori* hypotheses, rather than a single family of hypotheses requiring family-wise error correction. Regardless, we used an alpha value of 0.01 for significance testing throughout this study.

For the bootstrap test, the initial data matrix is resampled with replacement within each condition. For the contrasts we present here, we plotted the *task latent variables*, together with the mean brain scores and 95% confidence intervals derived from the bootstrap test after mean-centering and normalizing with the respective singular value. Two conditions can be reliably distinguished by a contrast if one's confidence interval does not include the mean of the other. For the saliences of *brain latent variables*, we calculated bootstrap ratios by dividing each element by the standard error of its corresponding bootstrap-sample distribution. Bootstrap ratios greater than 2.5758 approximate the 99th two-tailed percentile for a particular element (see Z-score table). In the following, we primarily interpreted data points above this threshold, i.e. the most extreme values, and therefore those that have the highest impact on the LV. However, the LV is represented by *all* element saliences. For each of our two measures (*WP* and *PLI*), we ran two separate PLS analyses corresponding to our two major hypotheses: one comparing FEEs with natural dynamics to those with unnatural dynamics, and one comparing those with natural dynamics to static images with no dynamics. Thus, each of the PLS analyses included a 2 (EXPRESSION) × 2 (MOTION) condition design (2 x 2–1 = 3 degrees of freedom). Both analyses included the two EXPRESSION levels, *happy* and *angry*, but each included only two of the three MOTION levels: *natural*, and *unnatural*, indicating the type of dynamics in the displayed FEEs. In particular, the first PLS included the MOTION levels *natural* and *static*, i.e., a comparison that has been studied in the neuroimaging literature in the past raising questions about the causes of the observed enhanced brain activations to dynamic FEEs (see Introduction for details); the second PLS included the MOTION levels *natural* and *unnatural* and allowed us to study the effect of dynamics on brain processing of FEEs controlling for the total amount of movement. Splitting the analysis into two separate tests permitted us to test each hypothesis in isolation and avoided potentially difficult-to-interpret interactions among the conditions. All PLS analyses returned 4 LVs, the last of which did not explain any covariance, since the degrees of freedom of all designs were 3.

### Analysis of behavioral data

**Fig 1:**
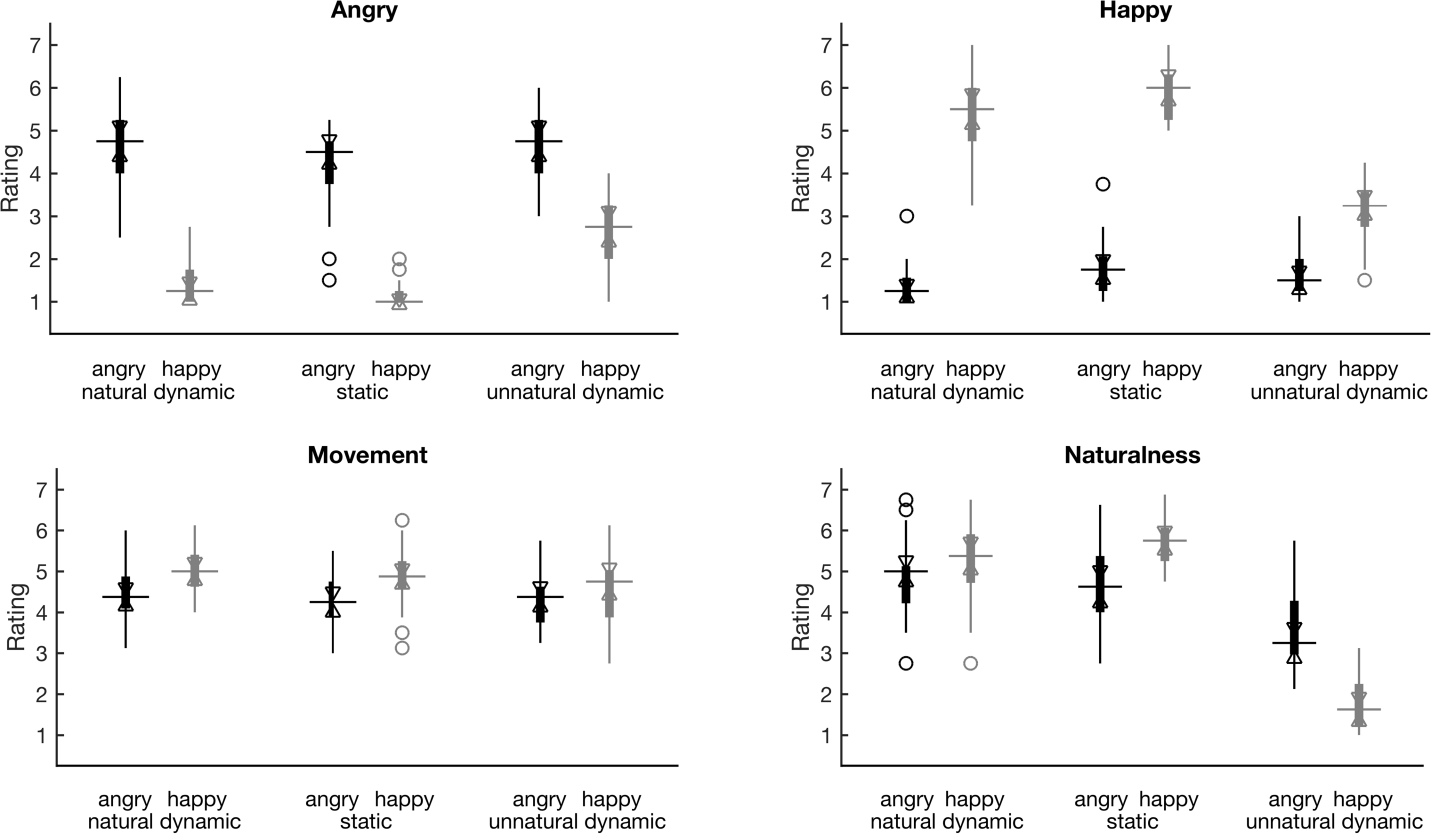
Stimuli’s validation ratings. Ratings shown are of “Angry” (top left), “Happy” (top right), “Movement” (average of the ratings for the amount of eyes and mouth movement; bottom left), “Naturalness” (average of the two related ratings; bottom right). The ratings for the stimuli of *angry* (*happy*) expression are depicted in boxplots of black (grey) color, whereas both EXPRESSION and MOTION levels are shown along the *x* axes of the plots. The boxplots depict the median (*q*_2_, horizontal line) and the 25th and 75th percentiles (*q*_1_ and *q*_3_, respectively, vertical line whiskers), while non-filled triangle notches define intervals, the lack of overlap of which between any pair of variables, signals that the respective medians are significantly different at the 5% significance level [52] (see also Tables A-D of S1 File for pairwise comparisons). The notch extremes of those intervals correspond to $q_{2}\pm 1.57\left(q_{3}-q_{1}\right)/\sqrt{n}$, where *n* is the number of observations (participants in our case). Outliers are also shown as non-filled circles.

**Fig 2:**
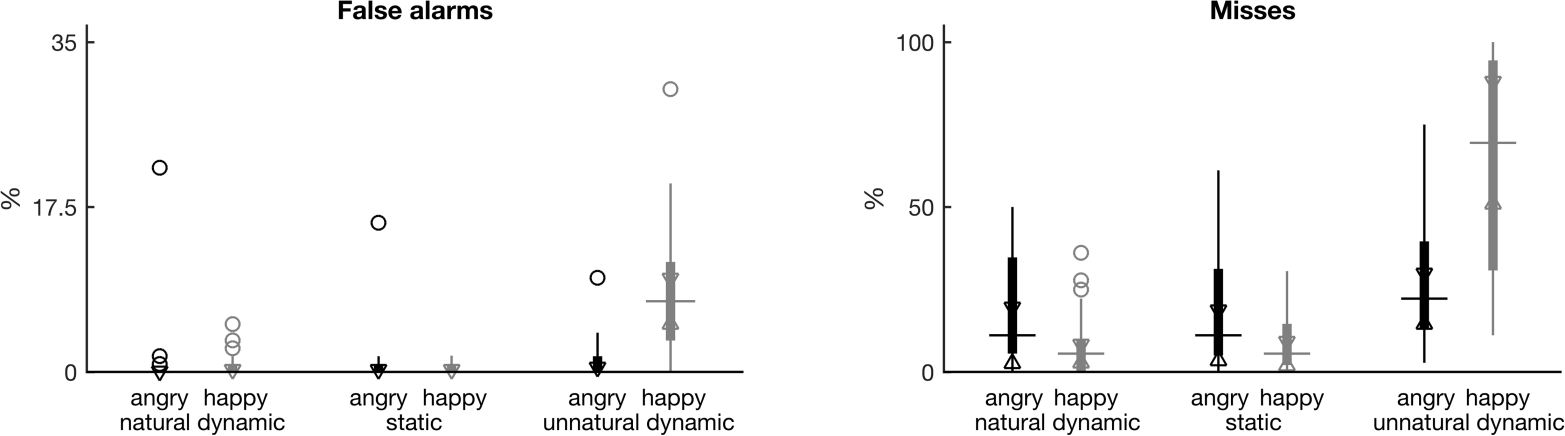
Mean error percentages per participant. Boxplots of subject means of false alarm (response although a standard stimulus was presented) and miss (no response although a deviant stimulus was presented) percentages are shown at the left (right) panel, respectively. Boxplot conventions and *x* axis description are identical to Fig 1. Note the different scaling of the *y*-axes. See also Tables E & F of S1 File for pairwise comparisons.

**Fig 3:**
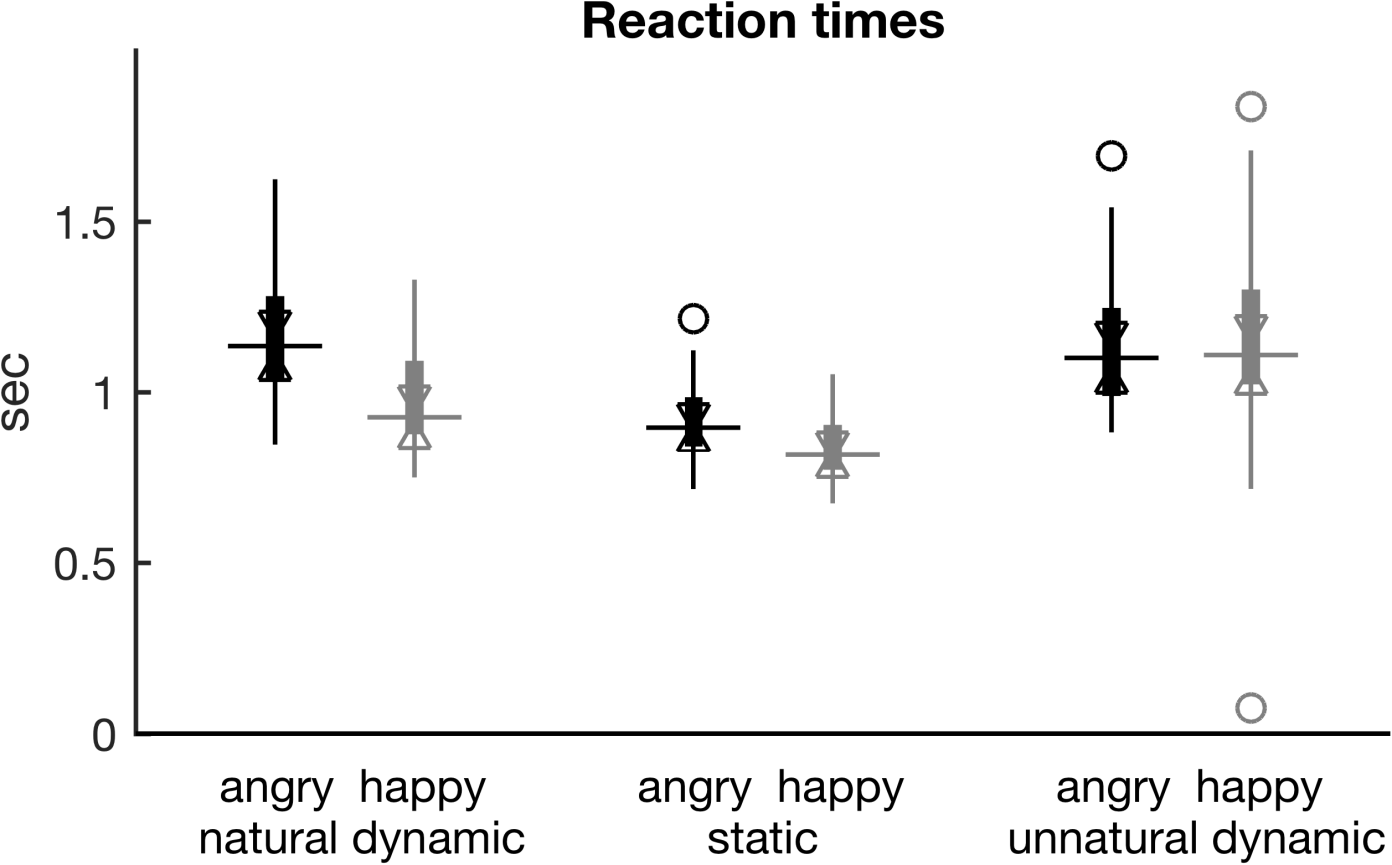
Reaction times. Boxplots of reaction times are shown. Boxplot conventions and *x* axis description are identical to Fig 1. See also Table G of S1 File for pairwise comparisons.

We tested the validation ratings of the stimuli, as well as error rates and reaction times from the main oddball task for the statistical effects of the two factors (EXPRESSION and MOTION). For validation ratings and error rates we performed a Friedman test, i.e., a non-parametric within-subject test, as implemented in the Statistics Toolbox (The MathWorks, Inc.). To be able to distinguish main and interaction effects, we ran an independent Friedman test for each effect (main effect of EXPRESSION, main effect of MOTION, and interaction effect of EXPRESSION × MOTION), and corrected for multiple testing by using a Bonferroni-corrected alpha-value of 0.017 (= 0.05/3). Subsequently, we performed post-hoc pairwise comparisons’ tests using Tukey’s honest significance difference correction (using *multcompare*.*m* function of MATLAB), the results of which can be viewed in Tables A-F of the S1 File. For the validation ratings, we performed the analysis for the variables "Angry”, "Happy”, "Movement” (the average of the two participant ratings measuring the amount of movement in the stimuli) and "Naturalness” (average of the two participant ratings measuring the naturalness of the stimuli). Reaction times were first log-transformed and then tested with a two-way repeated measures ANOVA in SPSS 20.0 (IBM Corp.). EXPRESSION and MOTION conditions were treated as within-subject factors and the alpha value was set to 0.01. Degrees of freedom were Greenhouse-Geisser corrected when necessary. The whole output of SPSS, including results for pairwise comparisons of marginal means with Bonferroni correction can be viewed in Table G of the S1 File. The behavioral data are presented in boxplots (Figs 1–3) constructed with the MATLAB function *boxplot*.*m*.

## Results

### Behavioral

The behavioral results show that stimulus construction was in general successful, with *unnatural* dynamic stimuli rated as less natural, and as having more ambivalent emotional content, even though the total amount of movement they contained was similar to *natural* stimuli (and perceived as such according to the related ratings). *Unnatural* dynamic stimuli also elicited slower reaction times than natural ones, an effect more prominent for the *happy* FEEs than for the *angry* ones. We expect that this is due to the larger amount of movement displayed in the original *natural happy* stimuli than in the *angry* ones (as indicated also in the participants’ related ratings), leading to some stimuli imbalance. More overall movement allowed for a more intricate distortion of the natural dynamics and consequently, of their emotional content. *Natural* dynamic and *static* stimuli did not differ substantially in their ratings or error rates.

#### Validation ratings

Visual inspection of the validation ratings boxplots (Fig 1, top left panel) shows that *angry* FEEs were rated as ‘Angry’ more than the *happy* FEEs. Indeed there was a significant main effect of EXPRESSION for the variable ‘Angry’ (χ^2^(1) = 360, *p* < 0.0001) according to the Friedman test. The inverse is true for the variable ‘Happy’, since *happy* FEEs received higher ratings (see Fig 1, top right panel), also leading to a significant main effect of EXPRESSION (χ^2^(1) = 425, *p* < 0.0001). There was no main effect of EXPRESSION for the variable of ‘Naturalness’ (Fig 1, bottom right panel; χ^2^(1) = 0.239, *p* = 0.625), unlike, unfortunately, for the variable of ‘Movement’ (Fig 1, bottom left panel; χ^2^(1) = 26, *p* < 0.0001), according to which *angry* stimuli were rated as containing less movement than *happy* ones, leading to a small stimuli imbalance. Regarding the main effect of MOTION, it was present for variables ‘Angry’ (χ^2^(2) = 43, *p* < 0.0001) and ‘Happy’ (χ^2^(2) = 37, *p* < 0.0001), indicating that the distortion of dynamics had indeed an effect on the perception of the emotional expression, as well as for the variable ‘Naturalness’ (χ^2^(2) = 226, *p* < 0.0001) as expected. However, the perturbation upon perception was more evident in the interaction effects of EXPRESSION x MOTION for the variables ‘Angry’ (χ^2^(5) = 422, *p* < 0.0001), ‘Happy’ (χ^2^(5) = 497, *p* < 0.0001) and ‘Naturalness’ (χ^2^(5) = 300, *p* < 0.0001), where the perception of *happy unnatural* stimuli is most strongly affected as Fig 1 suggests. At the same time, by visual inspection of the bottom right panel of Fig 1, we observe that these stimuli are also rated as less natural than the *angry unnatural* stimuli, in which case the stimuli imbalance is again present (see also Tables A-D of S1 File for pairwise comparisons). Finally, there was no main effect of MOTION for the variable ‘Movement’ (χ^2^(2) = 5.278, *p* = 0.071), whereas there was a weak MOTION and EXPRESSION interaction for the same variable (χ^2^(5) = 34, *p* < 0.0001).

#### Error rates

Visual inspection of the error rates (Fig 2) indicates that the ones of *happy unnatural* stimuli were higher than those of any other stimuli, both for false alarms and miss rates. Accordingly, the Friedman tests showed that the strongest effects for both variables were the interaction effects of EXPRESSION x MOTION (χ^2^(5) = 148, *p* < 0.0001 for false alarms and χ^2^(5) = 205, *p* < 0.0001 for miss rates). In addition, MOTION showed significant main effects for both types of errors (χ^2^(2) = 75, *p* < 0.0001 for false alarms and χ^2^(2) = 148, *p* < 0.0001 for miss rate), *unnatural* FEEs leading to more errors in accordance with the central concept of the experimental design. Instead, the EXPRESSION main effect was significant only for the false alarm rates (χ^2^(1) = 36, *p* < 0.0001, whereas χ^2^(1) = 0.07, *p* = 0.80 for miss rates), since *happy* stimuli generally led to more false alarm errors than *angry* ones. Finally, visual inspection shows that miss rates were generally higher than false alarm rates, which can be attributed to the fact that participants were instructed to respond only when they were highly confident (see also Tables E & F of S1 File for pairwise comparisons).

#### Reaction times

Repeated measures ANOVA on the logarithm of the reaction time data revealed a significant main effect of EXPRESSION (F(1,23) = 14.4, *p* < 0.001), a significant main effect of MOTION (F(1.55,35.6) = 122, *p* < 0.0001), and a significant interaction effect (F(1.13,26.1) = 18.8, *p* < 0.0001). Mean reaction times were fastest for *static* (891 ms), followed by *natural* (1090 ms), and finally *unnatural* stimuli (1205 ms). However, visual inspection of Fig 3 shows that the difference between *unnatural* and *natural* stimuli is prominent only for the *happy* ones. Similarly, reaction times tended to be faster for the *happy* compared to *angry* stimuli among *natural* and *static* stimuli, but not among *unnatural* ones, as Fig 3. suggests (see also Table G of S1 File for pairwise comparisons).

### EEG measures

**Fig 4:**
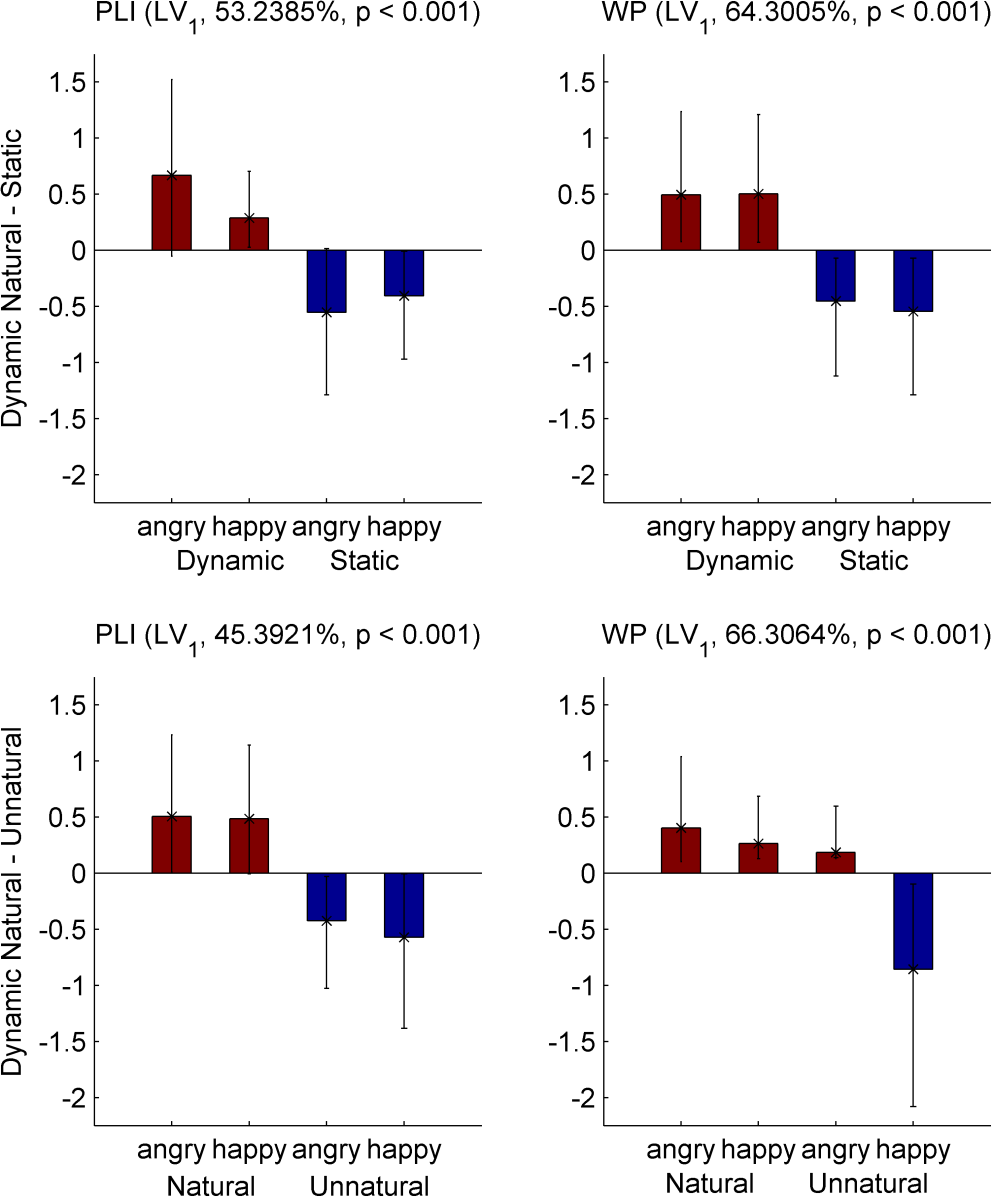
First task latent variable and normalized brain scores of EEG PLS analyses. Each panel shows the saliences of the first *task latent variable* (bars), and the normalized brain scores with 95% confidence intervals (asterisks and error bars) of the brain synchronization metrics. Top (bottom) panels depict the analyses of the *natural dynamic*—*static* (*dynamic natural*—*unnatural*) comparison (respectively), on *PLI* (left column) and *WP* (right column). The percentages of covariance explained and the *p*-values of significance are reported in parentheses for each contrast. Dark red (blue) bar color corresponds to the saliences of the natural dynamic (static or unnatural dynamic) level, respectively. Non-overlapping confidence intervals indicate that there are reliable differences among the corresponding levels.

**Fig 5:**
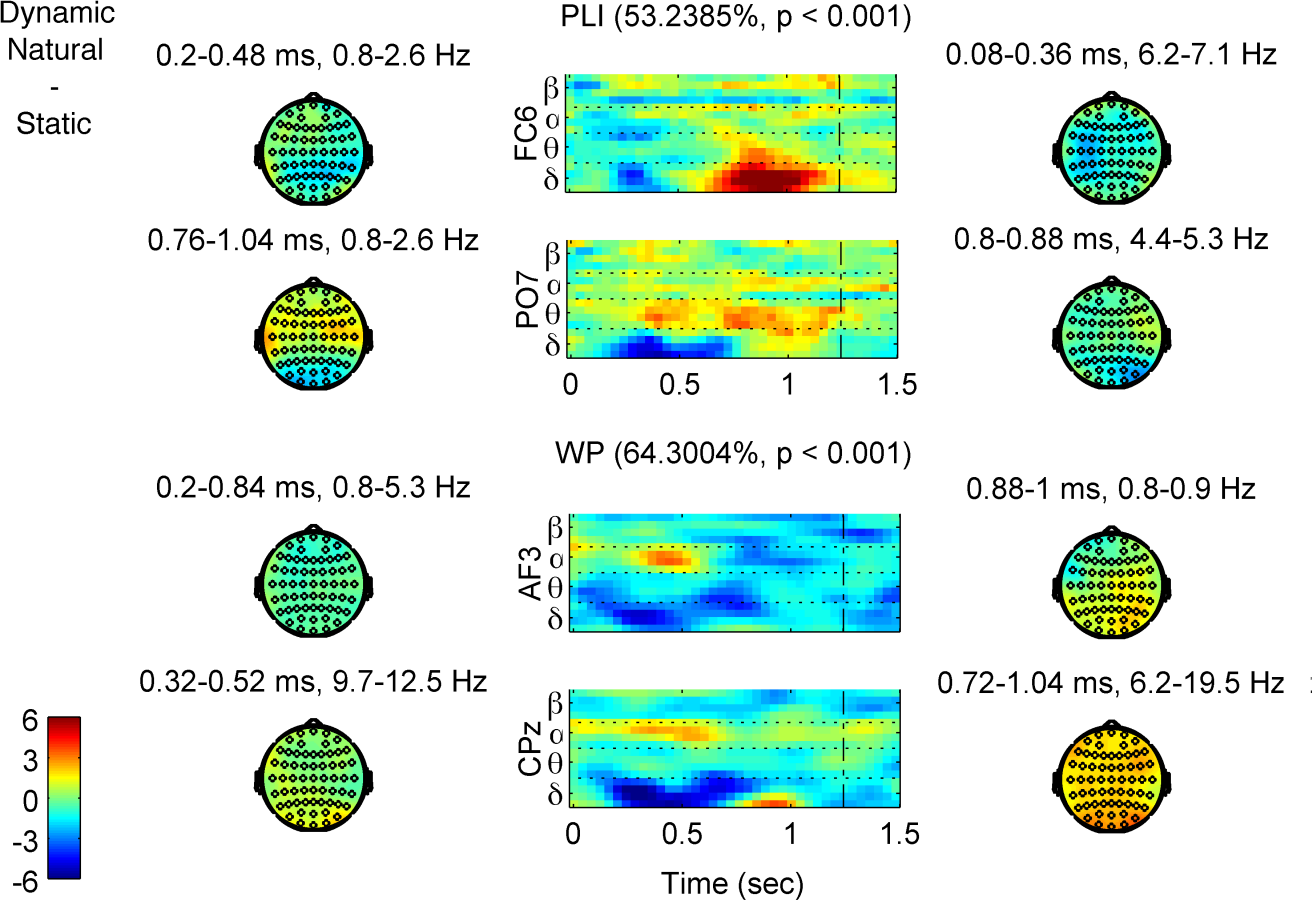
First brain latent variables (brain LVs) of the EEG PLS analyses for the Natural Dynamic–Static contrast. Time-frequency diagrams of characteristic channels (indicated at the y-axis label) and topographies of characteristic time-frequency windows of the element saliences’ bootstrap ratios for each contrast are shown. The percentages of covariance explained and the *p*-values of significance are reported in parentheses for each LV. Colors show how much each data *element*, i.e., a measure’s data point (PLI at upper two rows, and WP at the lower 2 rows), co-varies with the task contrasts of Fig 4 (top row) in terms of bootstrap ratios. So, red (blue) colors show higher (lower) values for the levels with positive (negative) task saliences of Fig 4 (top row), following the color bar at the bottom left corner. The (time-frequency-electrode) elements with the largest values are the most reliable in contributing to the contrasts. Time-frequency diagrams: the unit of the *x*-axis is milliseconds; the dotted vertical line indicates the end of stimulus; horizontal dotted lines separate frequency bands. Frequencies per frequency band are: delta: 0.9–3.6 Hz; theta: 4.4–7.1 Hz; alpha: 8.4–12.4 Hz; beta: 14.2–19.5 Hz. Topographies: distribution of bootstrap ratios of the elements’ saliences across the scalp averaged across a specific time-frequency window, as stated on top of the topography.

**Fig 6:**
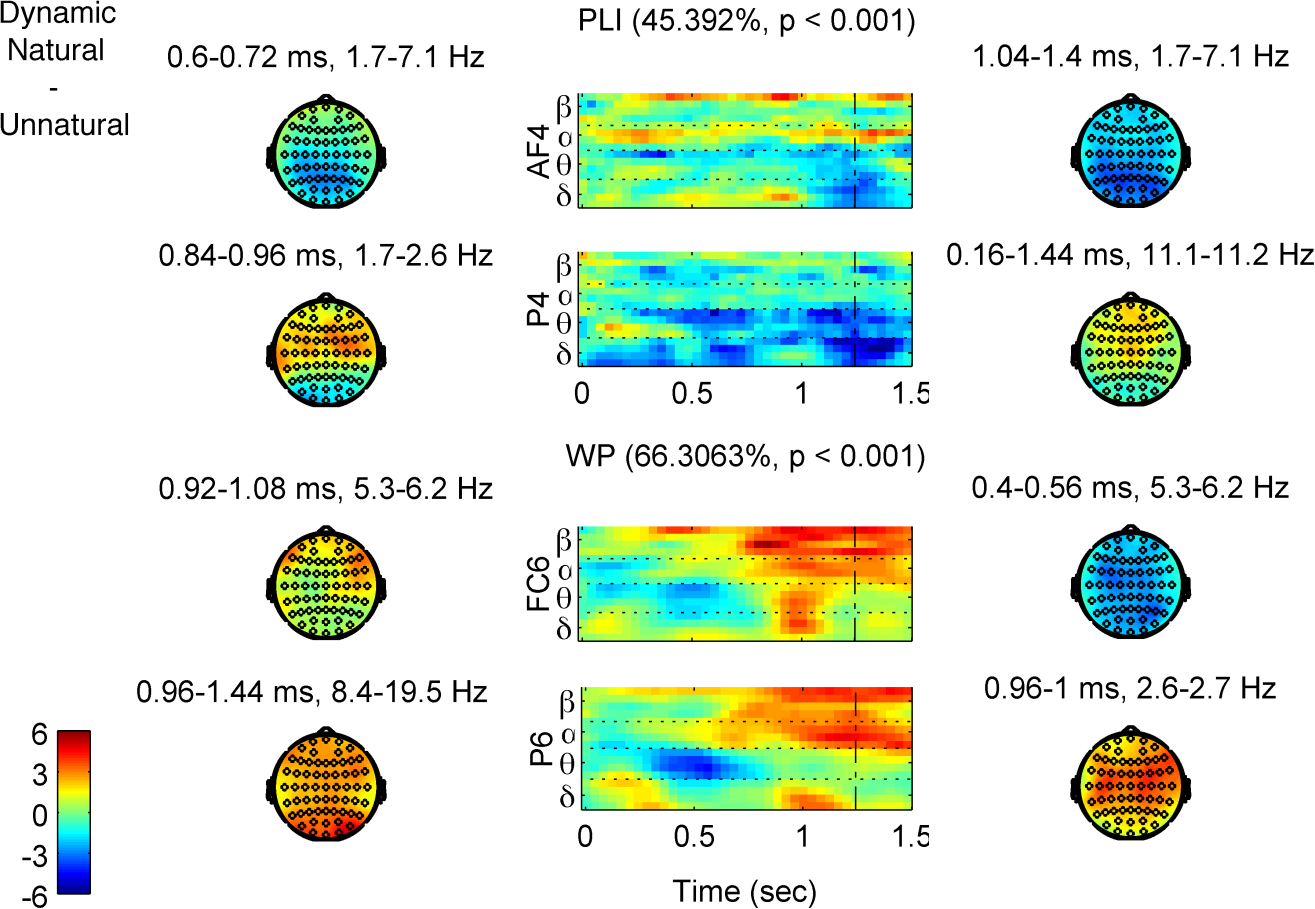
First brain latent variables (brain LVs) of the EEG PLS analyses for the Dynamic Natural–Unnatural contrast. The description of the figure is identical to the one of Fig 5, only now there is correspondence with the bottom row of Fig 4, i.e., with the comparison involving Natural and Unnatural Dynamics.

We ran in total four PLS analyses, two on each of our two measures *WP* and *PLI*. The first one included the *natural* and *static* levels of both EXPRESSION levels (*happy* and *angry*). The second PLS included the *natural* and *unnatural* MOTION levels, again of both EXPRESSION levels. Results of the permutation test for all significant LVs of each PLS analysis can be seen in Table 2. An alpha value for significance of 0.01 was used for all tests. Fig 4 depicts the task latent variables and the standardized brain scores of the PLS analyses, and Fig 5 & Fig 6 the respective brain latent variables of the two comparisons, whereas the *PLI* and *WP* time-frequency diagrams of the single conditions can be found in Figures C-H of the S1 File.

**Table 2:** Results of permutation tests for each PLS analysis and each LV.

	LV 1		LV 2		LV 3	
	p	r^2^	p	r^2^	p	r^2^
N vs. S						
*PLI*	<0.001	0.532	0.034	0.263	0.546	0.205
*WP*	<0.001	0.643	0.437	0.191	0.733	0.166
N vs. U						
*PLI*	<0.001	0.454	0.002	0.320	0.114	0.226
*WP*	<0.001	0.663	0.366	0.193	0.900	0.144

The fourth LV by definition accounts for zero covariance, because the degrees of freedom are 3, and is therefore not listed here.

#### Natural dynamic and static FEEs

Each one of the two PLS analyses with the *natural* dynamic and *static* FEEs on the measures *PLI* and *WP* revealed only a single significant LV (*p* < 0.001 for both), explaining 53.2% and 64.3% of the covariance respectively (Table 2). The confidence intervals of the standardized brain scores show that both LVs contrast the MOTION levels reliably (Fig 4, top row). The element saliences’ bootstrap ratios for both LVs (for *WP* and *PLI*) show that there was generally more processing of *static* FEEs at early latencies, and more prolonged processing of *natural* FEEs at later latencies. In particular, *PLI* (Fig 5, upper two rows, and Figure A, panel (a) in S1 File for a complete description) at delta band showed stronger phase locking for *static* between 150 and 700 ms (all times refer to post-stimulus onset times) over centro-parietal electrodes, whereas phase locking was stronger for *natural* after 700 ms over left fronto-temporal and right fronto-central sites, peaking around 900 ms. In the theta band, phase locking was stronger for *static* before 400 ms over fronto-temporal electrodes, but it was stronger for *natural* after 800 ms over right fronto-central sites (although the parieto-occipital electrodes showed higher theta *PLI* for *natural* consistently throughout the trial period). Accordingly, *WP* (Fig 5, lower two rows, and Figure A, panel (b) of the S1 File) followed the results of *PLI* at delta and theta bands, being higher at delta (over fronto-central sites) and theta (over bilateral temporal electrodes) bands for *static*, an effect that started as early as stimulus onset, and later (550 ms to 900 ms) moved into higher delta and lower theta bands and towards right fronto-centro-parietal sites. WP, by contrast, was higher for *natural* at lower delta band at later latencies, in particular, between 800 ms and 1000 ms (peaking around 900 ms), over centro-parieto-occipital areas (mostly right). In higher bands, there was stronger alpha desynchronization (*WP* decrease) for *static* before 500 ms over fronto-central (mostly left) and centro-parietal (mostly left) electrodes, which gave way to stronger upper theta, alpha and beta desynchronization for *natural* after 600 ms (peaking at 800 ms) over lateral occipital and fronto-temporal sites (stronger on the right); however, beta was desynchronized more for *static* at parieto-occipital sites throughout the trial period. Summarizing the results for this contrast, the processing of static FEEs exhibited stronger phase locking and power at earlier latencies, whereas the *natural* dynamic FEEs were processed during a longer period of time. This finding is to a large degree consistent across frequency bands (delta, theta, alpha and beta), with the exception of stronger theta phase locking, for *natural* dynamic FEEs and beta desynchronization for *static* FEEs, both of which were found at parieto-occipital sites and were sustained throughout the trial period.

#### Natural and unnatural dynamic FEEs

The PLS comparison of the *natural* and *unnatural* dynamic FEEs on the *PLI* measure revealed two significant LVs (Table 2). The first LV (*p* < 0.001) explains 45.4% of the covariance and contrasts the motion conditions (Fig 4, bottom left), whereas the second LV (*p* = 0.002) explains 32.0% of the covariance and captures an interaction effect between the EXPRESSION and MOTION conditions. Here we describe the element saliences’ bootstrap ratios only of the first LV (Fig 6, upper two rows, and Figure B, panel (a) of the S1 File) as the interaction is not directly related to our research questions and falls beyond the scope of the present work (readers interested in the interaction effect can see Figure B, panel (b) of the S1 File). The first LV shows that although phase locking distinguishes between *natural* and *unnatural* dynamic FEEs in distinct ways at different frequency bands, there is one prominent difference between anterior and posterior areas’ phase locking that is relatively consistent across frequencies. Specifically, *natural* FEEs show overall more phase locking in anterior areas, while *unnatural* FEEs show more in posterior areas. In particular, *PLI* at delta band is generally higher for *unnatural* at parieto-occipital sites (peaking around 650 ms), whereas it is higher for *natural* later on (after 650 ms), at fronto-centro-temporal sites (peaking around 800 ms over left temporal electrodes and around 900 over right fronto-central ones). Lower theta-band *PLI* is generally higher for *natural* at earlier times (peaking at parietal sites around 300 ms and at left frontal sites around 350 ms), whereas upper theta-band *PLI* is higher for *unnatural* at centro-parietal electrodes (with an early -around 250 ms- and a late–around 650 ms- peak). Note, also, that both delta and theta band show stronger phase locking for *unnatural* around the time of the end of stimulus and over widespread brain areas (after 1000 ms and more anterior for delta, after 900 ms and more posterior for theta). Finally, alpha- and beta- band *PLI* is generally higher for *natural* throughout the trial period, over fronto-centro-temporal sites (mainly right), this effect being stronger and more widespread for alpha than for beta.

Whereas all of the above PLS analyses revealed the strongest LV to clearly separate the MOTION levels, this is not true for the PLS comparison of *natural* and *unnatural* levels on the *WP* measure. The only significant LV (*p*<0.001), which explains 66.3% of the covariance, contrasts *happy unnatural* with the three other levels (Fig 4, bottom right, and Figure B panel (c) of the S1 File for a complete description) in the same way these levels are distinguished in terms of the behavioral error rates (Fig 2). The bootstrap ratios of the element saliences (Fig 6, lower two rows, and Figure B panel (c) of the S1 File) show generally lower *WP* for *happy unnatural* FEEs at all bands, mainly at later latencies, with the exception of an early opposite effect at delta and theta bands. In particular, *WP* at delta band was lower for *happy unnatural* at two time periods: an early one at 250–650 ms (peaking around 250 ms over right parieto-occipital sites, and at 550 ms over centro-parietal electrodes), and a later one at 900–1150 ms (peaking at 950 ms) over fronto-centro-temporal (more right) electrodes. With regards to theta band, *WP* was higher for *happy unnatural* at 250–550 ms, (peaking at 400 ms) over frontal (more left) and centro-parietal (more right) sites, but lower for *happy unnatural* later on, after 850 ms (peaking at 950 ms) over bilateral fronto-temporal sites. Last but not least, the most prominent effect appeared in the alpha and beta bands, which showed much lower *WP* for *happy unnatural*, towards late latencies (after 500 ms, peaking around 1200 ms) over widespread regions (more right).

## Discussion

The natural movement course of a FEE starts from an initial, emotionally “neutral” face and gradually develops to an emotional expression, following a highly nonlinear trajectory [29]. We aimed at learning more about how specific brain responses to FEEs are to this dynamic trajectory. To that end, we studied how these brain activations change when we stimulate the brain with static and unnatural dynamic FEEs, which each impair the dynamics in distinct ways. Specifically, we analyzed EEG measures of local brain synchrony and phase locking with high time and frequency resolution, by employing “mean-centering task PLS”, a data-driven multivariate method that finds contrasts between conditions in a way that maximizes differences in neural response. The manipulation was successful in that three of the four PLS analyses found the maximal contrast to be between motion types. However, the fourth PLS analysis revealed the maximal contrast not between motion levels, but between levels with high and low task difficulties, as indicated by behavioral error rates and participant reports. Our results showed that the way the brain processes a FEE depends on the stimulus’ dynamics in a complex manner that shows qualitative differences above and beyond the additive influences of total variability, movement, or quantity of task-specific information in the stimuli. In the remaining part of the Discussion, we first discuss briefly the implications of the behavioral results for the interpretation of the EEG analysis. We then discuss some aspects of our most prominent results from the perspective of how the impaired dynamics of FEEs (either static or unnatural dynamic) differentiates the brain processing of FEEs of natural dynamics.

### FEEs’ movement dynamics affect the perception of emotions

Participant ratings of the stimuli validated their emotional content and confirmed that the construction of unnatural dynamic stimuli of equal amount of movement with the natural dynamic stimuli was successful. At the same time, the ratings of “Movement” showed that happy stimuli were perceived as depicting a stronger movement than the angry stimuli. Emotional ratings are in accordance with error rates and reaction times in revealing that the happy and angry stimuli were also not matched in task difficulty. In particular, the emotional content of happy natural dynamic and static FEEs was more easily accessed than that of the corresponding angry ones, a relationship that reversed for the unnatural dynamic stimuli. These results, considered together, confirm the importance of the natural dynamics for the perception of FEEs, since the strong natural movement of the happy stimuli facilitates perception, and its distortion impedes it, whereas the weaker natural movement in the angry stimuli reduced the effects of this distortion. Our behavioral results are in accordance with the studies mentioned in the introduction that managed to impede the perception of emotion by distorting the dynamics of FEEs through time reversal [29], frame scrambling [16,18,29] or masking [14]. Unlike in the studies of [12–16], however, we did not find any significant improvement of perception for natural dynamic stimuli compared with static ones in any of validation ratings, error rates or reaction times. The fact that the emotion-specific information of our natural dynamic stimuli is presented at later latencies might explain why reaction times are faster for the static stimuli. With respect to error rates, the lack of any perceptual improvement could be due to the fact that the happy (or angry) natural dynamic stimuli were more likely to be confused with the unnatural dynamic angry (or happy) stimuli (respectively) than were static images.

### Differences in the timing of brain processing of natural dynamic versus static FEEs

Unlike natural dynamic FEEs that develop gradually from an emotionally “neutral” expression to an expressive peak, static FEEs present only that peak, right at stimulus’ onset and invariantly throughout a trial’s duration. In our opinion, this difference in the timing of presentation of the task specific information is revealed in the following results that differ between early and late latencies: delta phase locking (higher *PLI* for *static* before 650 ms and for *natural* after 700 ms), theta phase locking (higher *PLI* for *static* before 400 ms and for *natural* after 750 ms), delta and lower theta power (higher *WP* for *static* within 100–900 ms and for *natural* within 750–1050 ms), and alpha desynchronization (lower *WP* for *static* before 500 ms and for *natural* after 650 ms). By considering them together, these results show that *static* stimuli were processed more strongly at early latencies, whereas *natural* dynamic stimuli entailed more prolonged processing towards later latencies as expected. (Note that the delta and lower theta *PLI* response to the *static* -unlike the *natural*—FEEs is back to baseline after 700 ms–see Figure A panel (a) of the S1 File) This conclusion agrees with previous studies [22,28] and supports the hypothesis of [19] that prolonged rather than stronger activations could explain the stronger fMRI and PET responses to dynamic FEEs over their static counterparts [17–20]. Indeed, *PLI* and, even more, *WP* at low frequencies are far from showing stronger activations for *natural* dynamic than for *static* stimuli in our results, especially before 800 ms. The only exception is the effect of stronger theta phase locking for *natural* FEEs at parieto-occipital sites, which is present throughout a trial’s duration. This effect could be a result of activity at visual areas driven by the varying dynamic stimuli against the lack of such activity for the static ones.

### Differences in brain measures of processing dynamic natural versus unnatural FEEs that relate to the difficulty to recognize their emotional content

Unnatural dynamic FEEs present emotion-related information in a non-coherent, unpredictable manner throughout a trial’s duration. Such a movement, although biologically plausible, is atypical compared to ecologically-validated FEEs. In comparison to the perception of natural dynamic FEEs, participants take longer to process the visual input and encounter increased difficulty linking it with existing past experiences and memories in order to decode it. An analogy of this process could be the strenuous effort of trying several wrong keys, one after the other, in order to open a locked door, as compared to the more natural movement flow of opening the door with the correct key. We propose that such a process is reflected in our results. In particular, higher delta and theta phase locking for *unnatural* at parieto-occipital areas, especially after 500 ms post-stimulus’ onset, could be indicative of repetitive phase resetting to the unnatural dynamics of the stimulus in a process of sampling task specific information. In contrast, frontal electrodes generally exhibit stronger delta and theta phase locking to the stimulus for *natural* (except for a time window around the end of stimulus), a result that could be a correlate of processing the coherent and predictable trajectories of natural dynamic FEEs.

The increased task difficulty of recognizing happy unnatural dynamic FEEs and of deciding whether to respond or not, may also be reflected in the alpha and beta *WP* after 500 ms. Alpha and low-beta desynchronization has been associated with general task demands and complexity, attentional processes including alertness during emotional face elaboration [39], as well as with effort and more efficient performance in cognitive and memory tasks [53], including the processing of human facial expression [54] (for a nice overview see [34]). In addition, the so-called *mu* rhythm (alpha and low beta rhythm over sensorimotor areas) has been linked to motor imagery during biological motion observation [55], including viewing emotional faces [56]. Last but not least, beta desynchronization has been associated with categorical action planning [57], and response selection [58] and uncertainty [59].

### Differences in the perception of FEEs of natural and impaired dynamics, either static or unnatural dynamic

Considering the results of both comparisons together, we observe that most of the effects that relate directly to the stimuli’s dynamics are found at low frequencies, i.e., delta and, to a lesser extent, theta phase locking (mainly) and power. Such effects are the early (for *static*) versus late (for *natural*) higher delta and theta *PLI* and *WP*, and the stronger parieto-occipital theta *PLI* for *natural* in the *natural*-*static* comparison, as well as the stronger delta and theta *PLI* at parieto-occipital areas for *unnatural* against the stronger phase locking at the same frequencies and at frontal areas for *natural*, in the *natural-unnatural* comparison. Moreover, focusing on the responses at those frequencies (delta and low -mainly- theta) in the time interval 800–1100 ms, one observes that natural dynamic stimuli are generally associated with increased phase locking and power. We suggest that at that time-frequency window, the brain processes stimulus information specific to the task of recognizing the FEEs’ emotional content, as proposed by Balconi and collaborators [39,40]. Last but not least, the topographies of those activations are generally fronto-centro-temporal with right hemisphere dominance, i.e., close to brain areas that have been associated with the perception of FEEs [10,21]. In contrast, the topographies of the respective windows of higher delta theta phase locking and power for static and unnatural dynamic stimuli are more posterior (centro-parietal). These observations agree with the suggestion [16] that FEEs with natural, coherent and predictable trajectories "are more easily" integrated at more anterior areas than unnatural FEEs (see also [60]).

### Limitations and future work

The most severe limitation of the present study concerns the experimental design, namely the lack of a control condition of non-biological and non-social or non-emotional nature, but with similar perturbations to the dynamics of motion, as in [61]. This is particularly important in light of the study of the Furl et al. [16], which stressed the sensitivity of the brain processes underlying FEEs’ perception on coherent and predictable movement trajectories. Such a control condition could allow us to further identify the biological, social or emotional specificity of the associated brain processes.

Another limitation of the study concerns the imbalance between the happy and angry stimuli, which were not matched in terms of the amount of movement and of task difficulty. Future work using more advanced approaches to facial expression modeling could allow us to generate facial emotional expressions that follow more precisely controlled trajectories both in movement trajectory and across emotional space [62]. Our choice of EEG as the primary measure of neural process, and of local neural population synchronization as a measure of outcome are naturally limited as well, with the the usual limitations in terms of spatial resolution and imprecision. Finally, the current study selected an entirely female sample, and the generalizability of the findings to males remains to be confirmed.

In future work, functional and/or effective connectivity analysis, including investigation for cross-frequency interactions, mainly between higher (e.g., alpha and beta) and lower (e.g., delta and theta) frequencies, given the present results, (but see also [63]), could reveal more about the brain networks that dynamically emerge to support the perception of natural FEEs. This analysis, performed in the EEG source space, could also help to place the study better in the context of the related fMRI or PET studies, as well as provide a stronger test of lateralization effects discussed in the literature [64]. Finally, single trial analysis could also make it possible to study in more detail the relationship between FEEs’ movement trajectories and the trajectories of brain responses.

### Conclusions

The present study used measures of amplitude (*WP*) and phase (*PLI*) of local brain oscillations to compare FEEs that differ in the naturalness of the movement dynamics. The results show that oscillatory brain activity discriminates among three classes of facial expression stimuli: FEEs with natural dynamics, FEEs with unnatural dynamics, and still images. Differences were observed in the timing and brain topographies of delta and theta PLI and WP, and in alpha and beta WP, i.e., both in the time and space components of the associated brain responses. On the one hand, our results cannot yet provide a thorough description of the brain processes underlying FEE perception (although they do support hypotheses made in [16]), partly due to limitations in the experimental and stimuli design. On the other hand, the observed differences in the brain spatiotemporal patterns cannot be reduced to differences in the total “quantity” of any characteristic, such as stimulus variability, movement, task-specific information contained in the stimuli, resulting task difficulty or required attentional demands. Instead, our results support the idea that the brain processes underlying FEEs’ perception are specific and sensitive to the natural dynamics of FEEs or, in other words, to the respective movement coordination as it has evolved biologically and in social practice. In particular, when the dynamics of FEEs is perturbed in a biologically plausible but socially atypical manner, the brain responses exhibit a complex pattern of spatiotemporal changes along with highly hindered perception. This conclusion is strengthened by the biological plausibility of our unnatural dynamic stimuli, which suppresses alternative possible explanations. Therefore, our results suggest more attention to be given to the ecological validity of stimuli employed in related experimental designs.

## Supporting information

S1 File Ensemble of supporting information figures and tables. It contains supporting information Figures A-H, and Tables A-G as referred to in the text.
(PDF)

S1 Video Natural dynamic happy expression of one of the actresses. (MOV)

S2 Video Natural static happy expression of one of the actresses. (MOV)

S3 Video Unnatural dynamic happy expression of one of the actresses. (MOV)
