# Supplementary material for: Brain synchronization during perception of facial emotional expressions with natural and unnatural dynamics
Source: PLoS One. 2017 Jul 19;12(7):e0181225. doi: 10.1371/journal.pone.0181225 (PMC5517022; doi:10.1371/journal.pone.0181225)
Supplement: S1 File — It contains supporting information Figures A-H, and Tables A-G as referred to in the text. (PDF) [file pone.0181225.s001.pdf]

Figure A.

Topography with *PLI* (a) and *WP* (b) time-frequency diagrams (21 electrodes of the 10-20 international system) depicting the bootstrap ratios of the element saliences of the 1st PLS LV for the comparison Natural Dynamic with Static. The respective brain scores' contrasts are shown as bar graphs at the bottom left corners of each plot.

a)

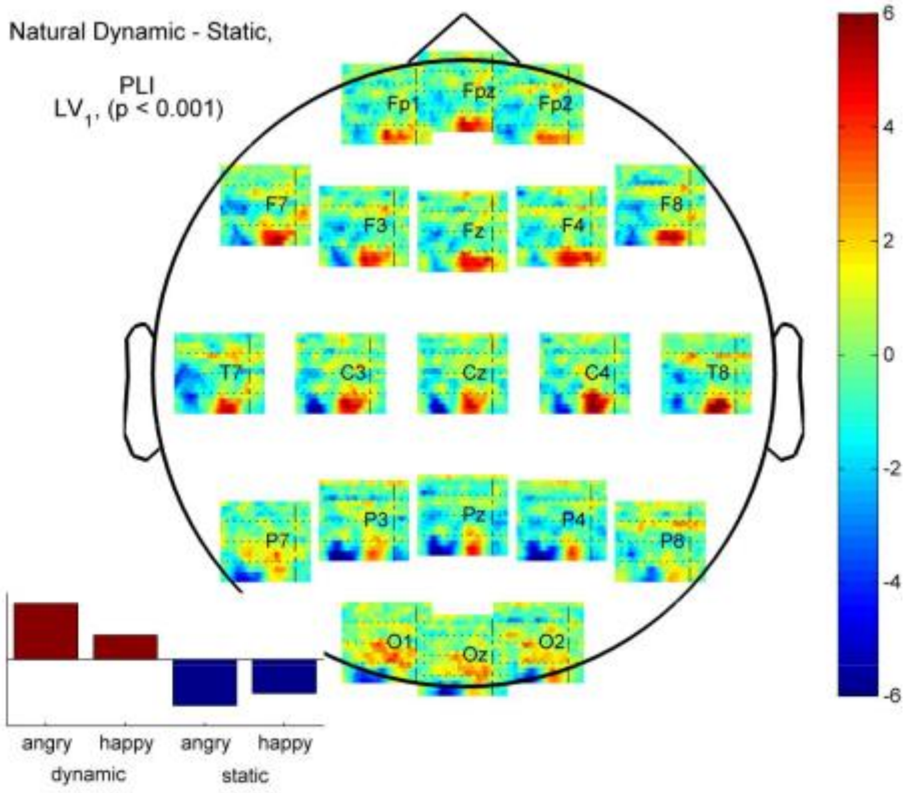


b)

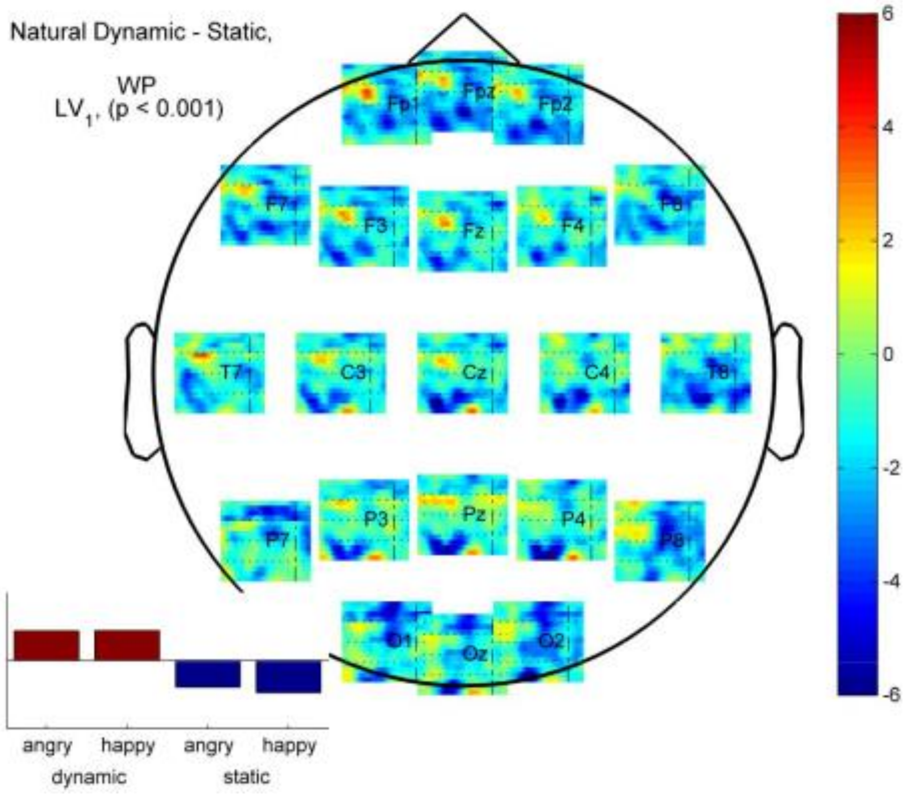

Figure B.

Topography with *PLI* (a) and *WP* (c) time-frequency diagrams (21 electrodes of the 10-20 international system) depicting the bootstrap ratios of the element saliences of the 1st PLS LV for the comparison Natural Dynamic with Dynamic Unnatural. (b) shows the 2nd significant LV for PLI describing the interaction effect between the MOTION and EXPRESSION factors. The respective brain scores' contrasts are shown as bar graphs at the bottom left corners of each plot.

a)

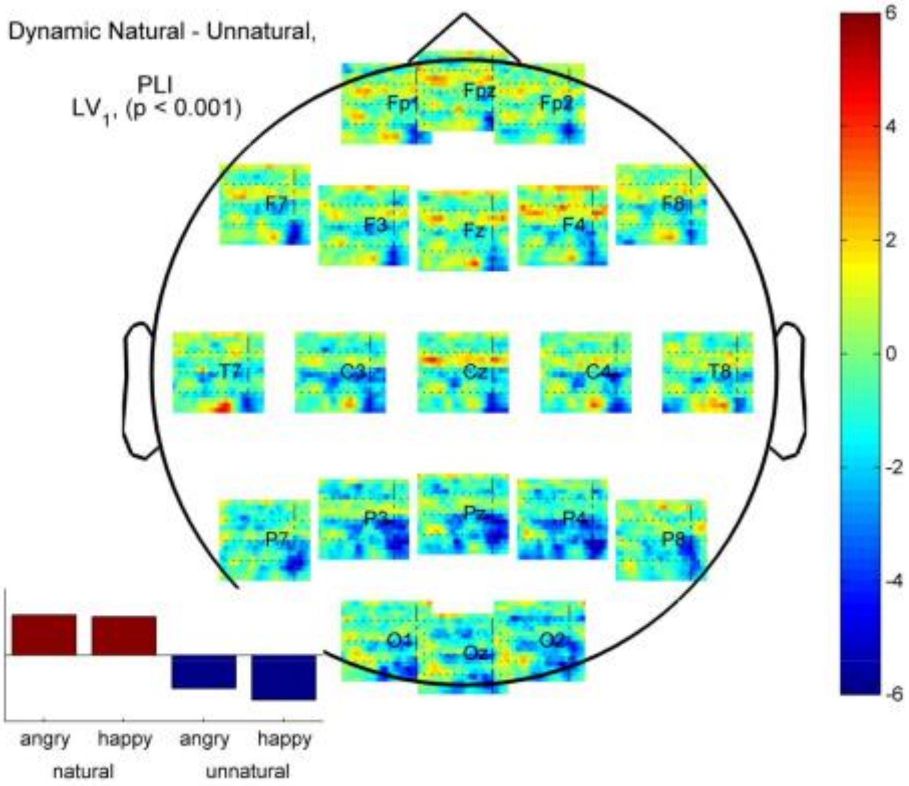


b)

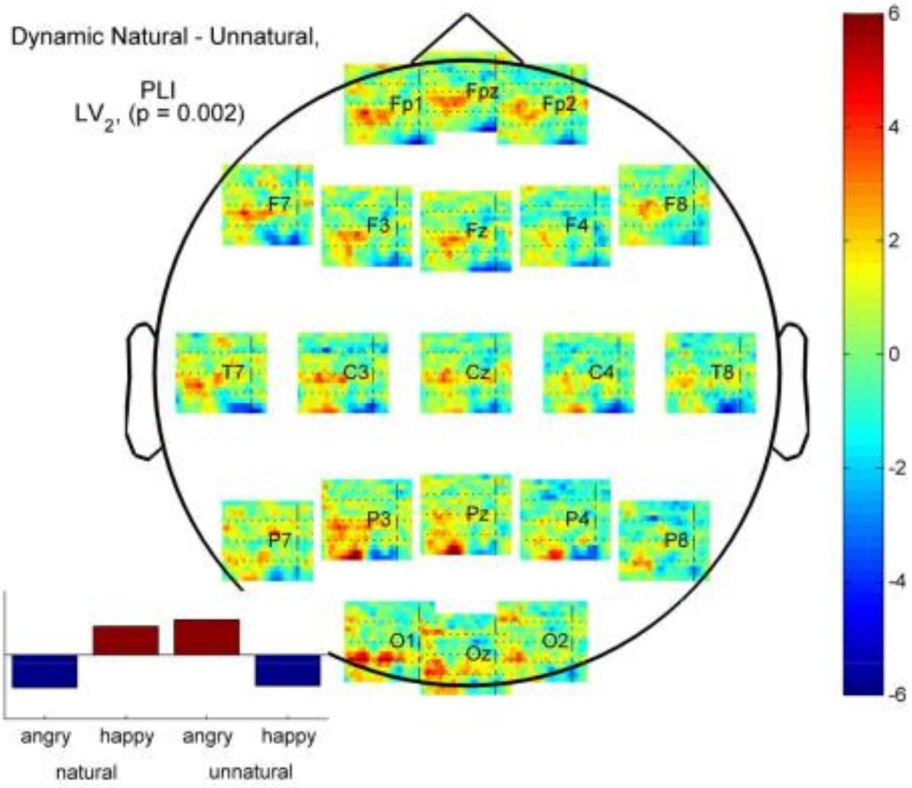

c)

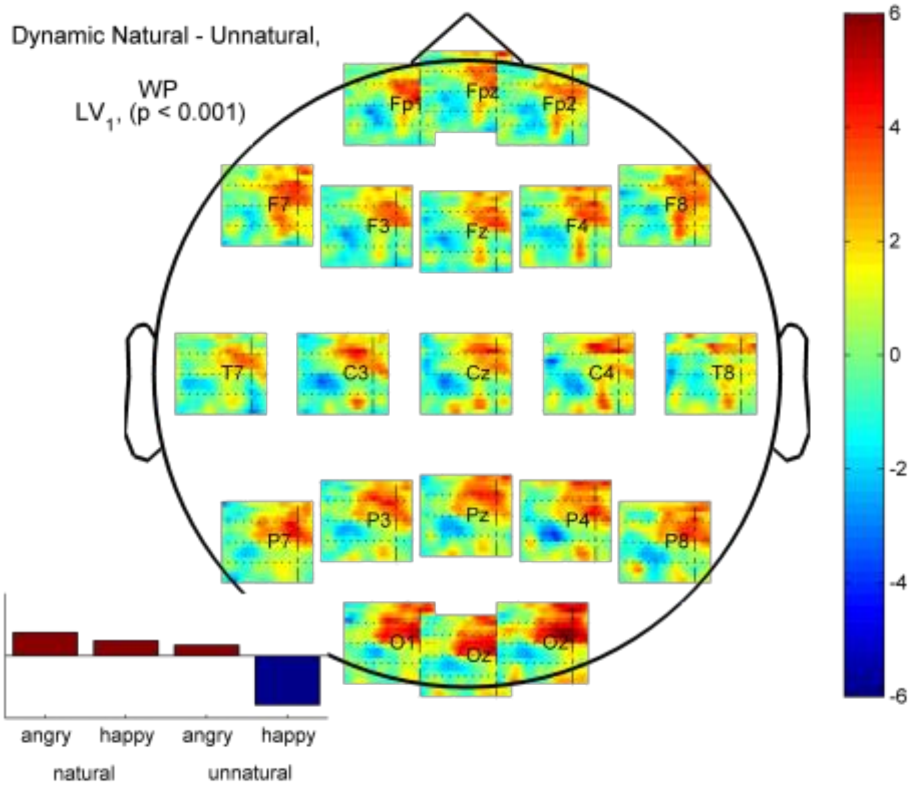

Dynamic Natural - Unnatural,
WP
LV1, (p < 0.001)
Fp1
Fpz
Fp2
F7
F3
Fz
F4
F8
T7
C3
Cz
C4
T8
P7
P3
Pz
P4
P8
O1
Oz
O2
6
4
2
0
-2
-4
-6
angry
happy
angry
happy
natural
unnatural

Figure C.

Topography with *PLI* (a) and *WP* (b) time-frequency diagrams (21 electrodes of the 10-20 international system) of the Angry Natural Dynamic condition.

a)

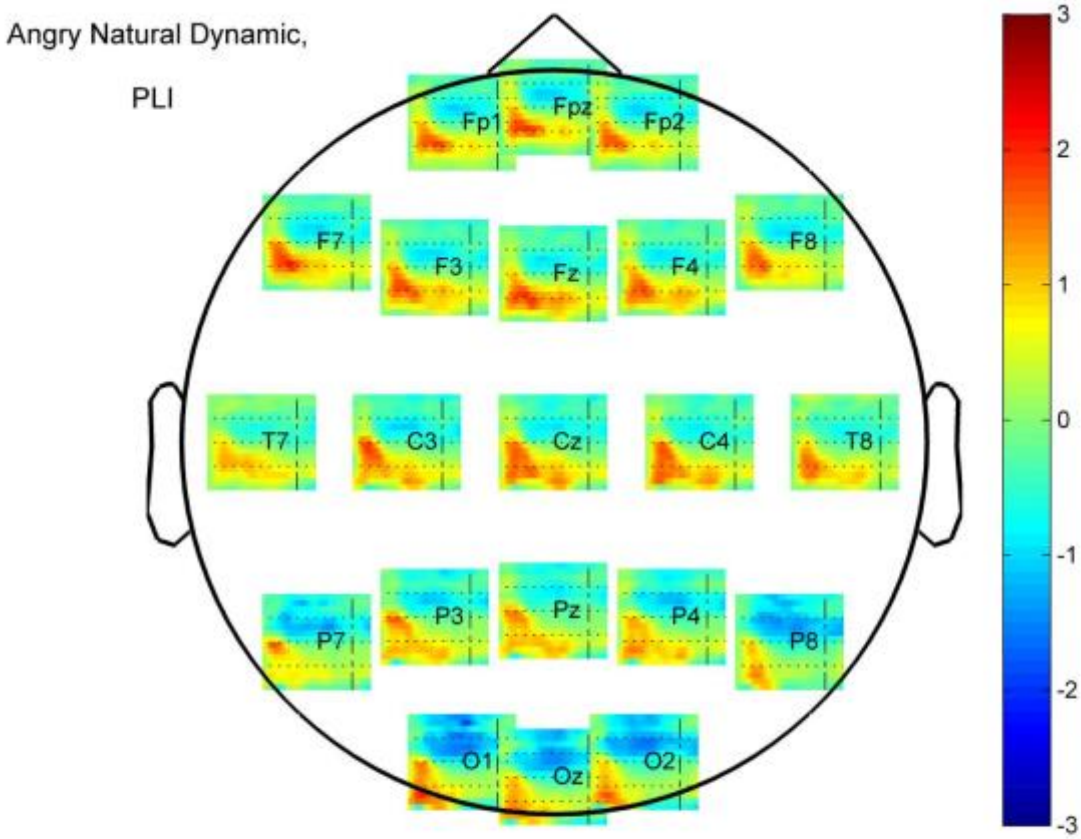


b)

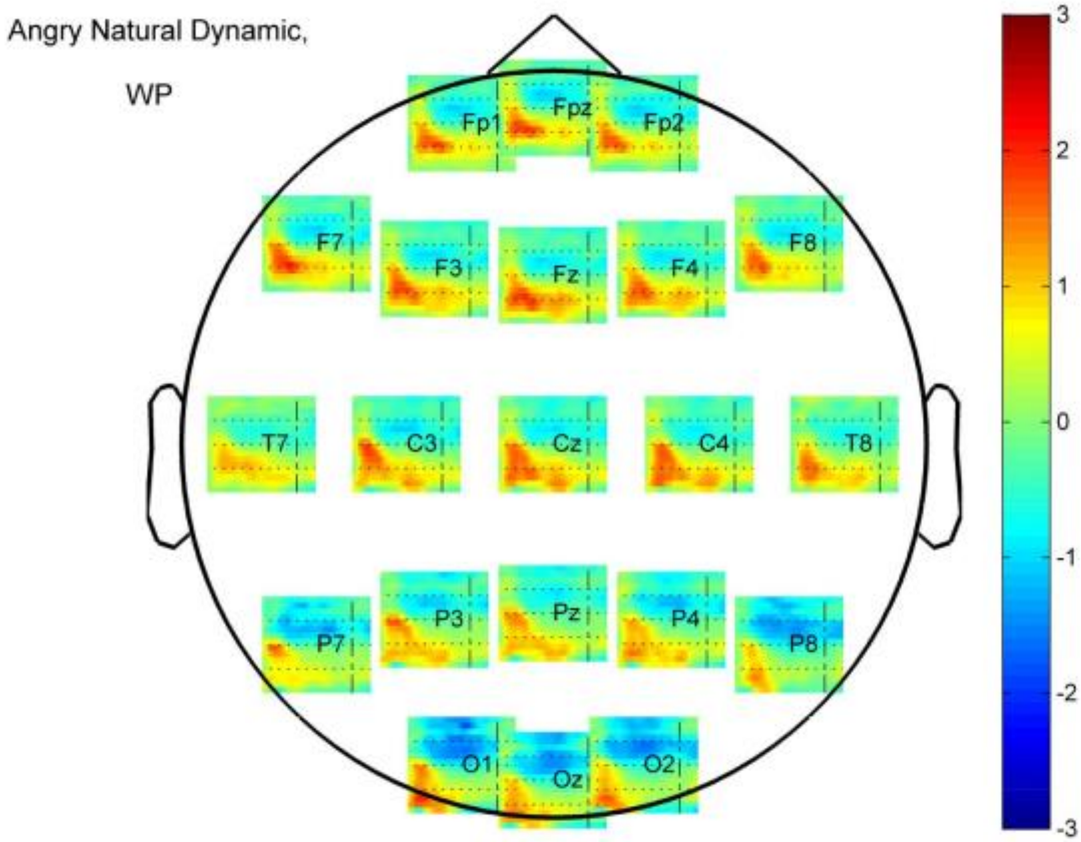

Figure D.

Topography with *PLI* (a) and *WP* (b) time-frequency diagrams (21 electrodes of the 10-20 international system) of the Happy Natural Dynamic condition.

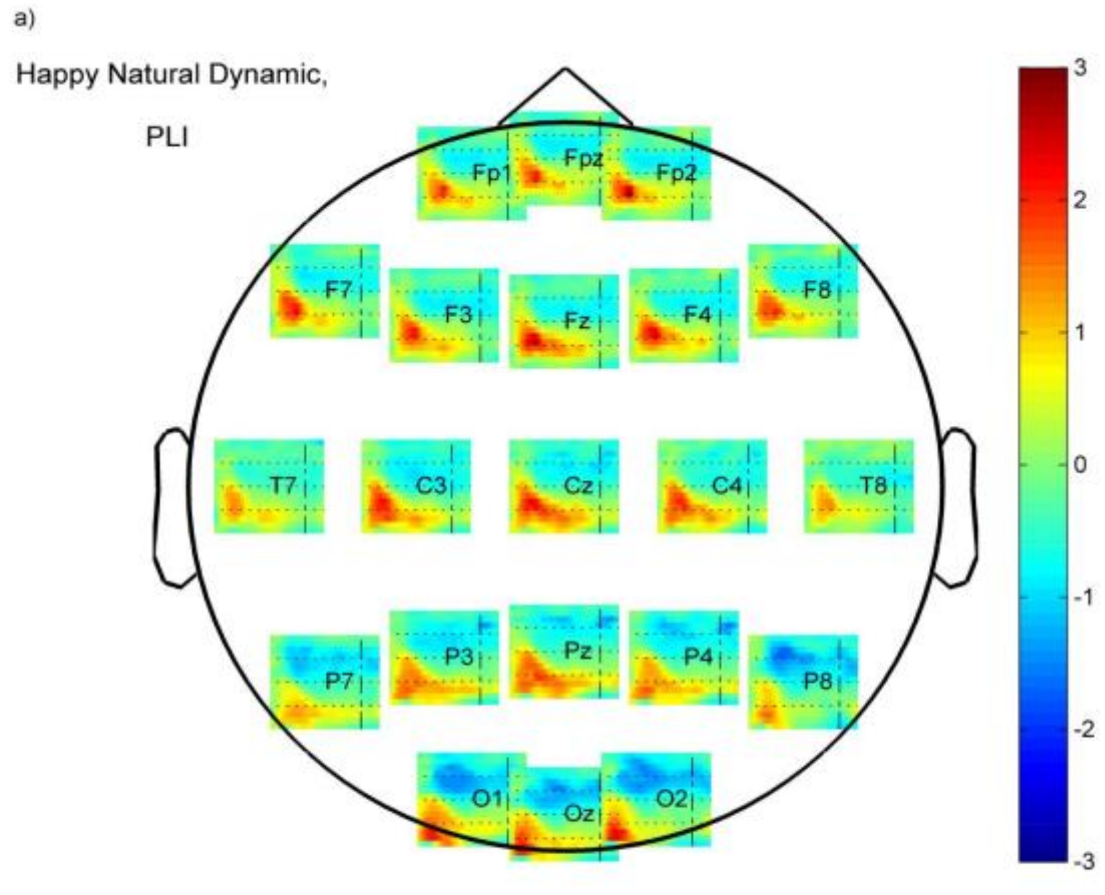


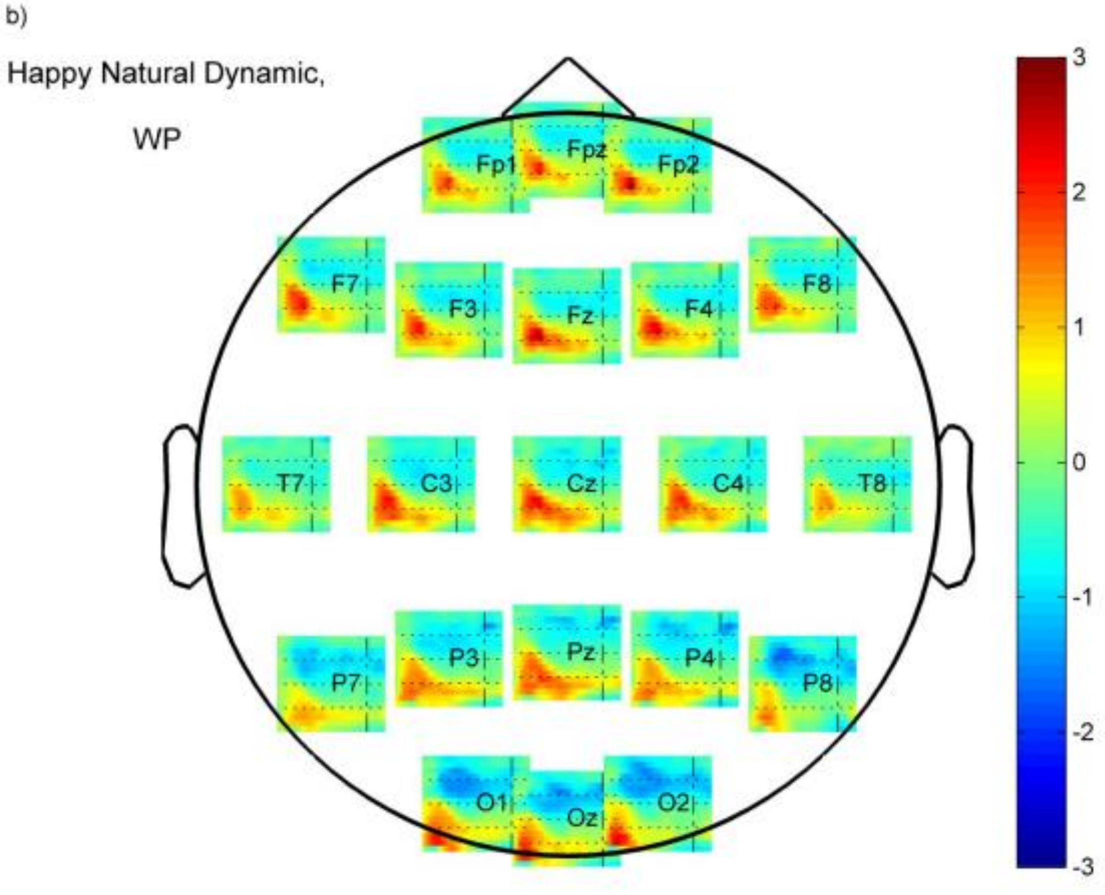

Figure E.

Topography with *PLI* (a) and *WP* (b) time-frequency diagrams (21 electrodes of the 10-20 international system) of the Angry Static condition.

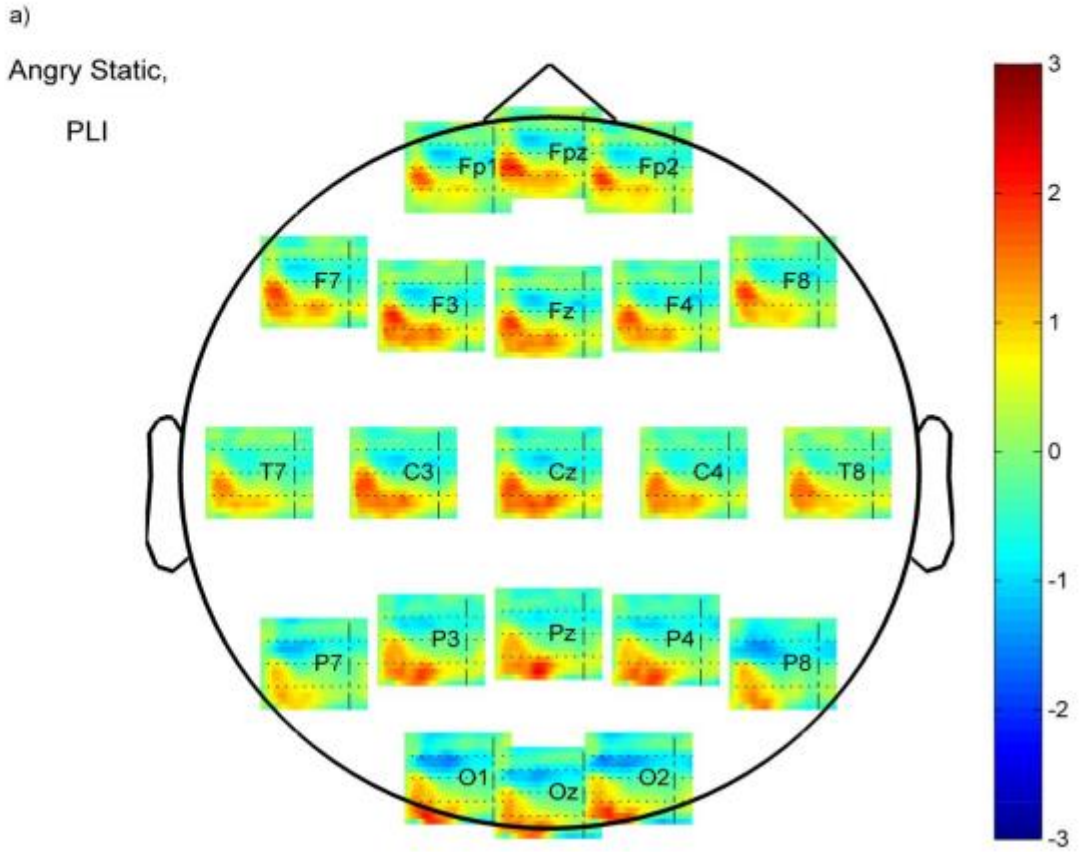


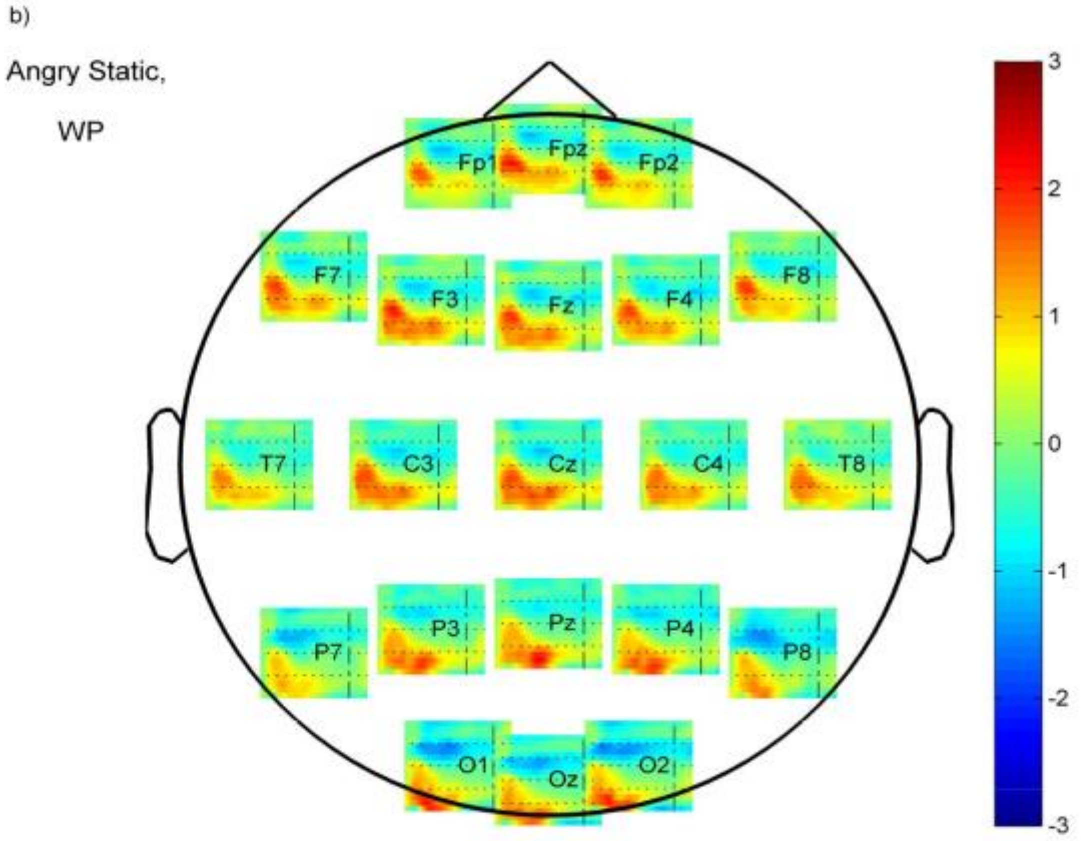

Figure F.

Topography with *PLI* (a) and *WP* (b) time-frequency diagrams (21 electrodes of the 10-20 international system) of the Happy Static condition.

a)

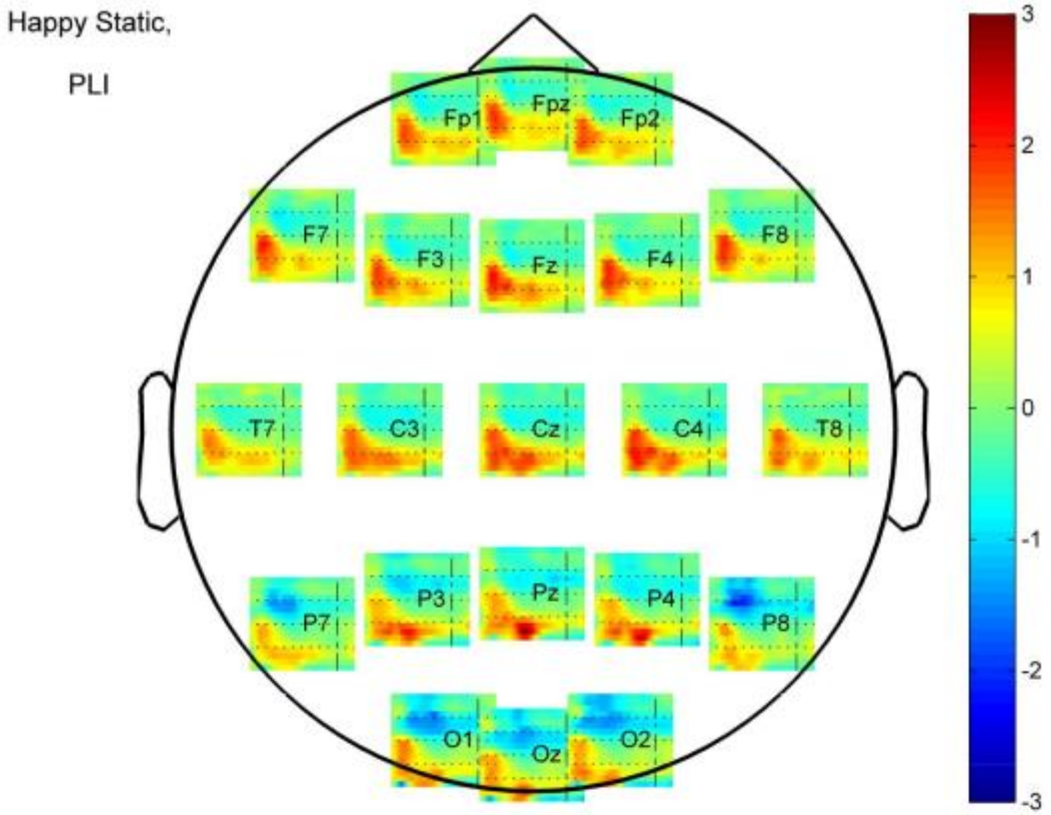


b)

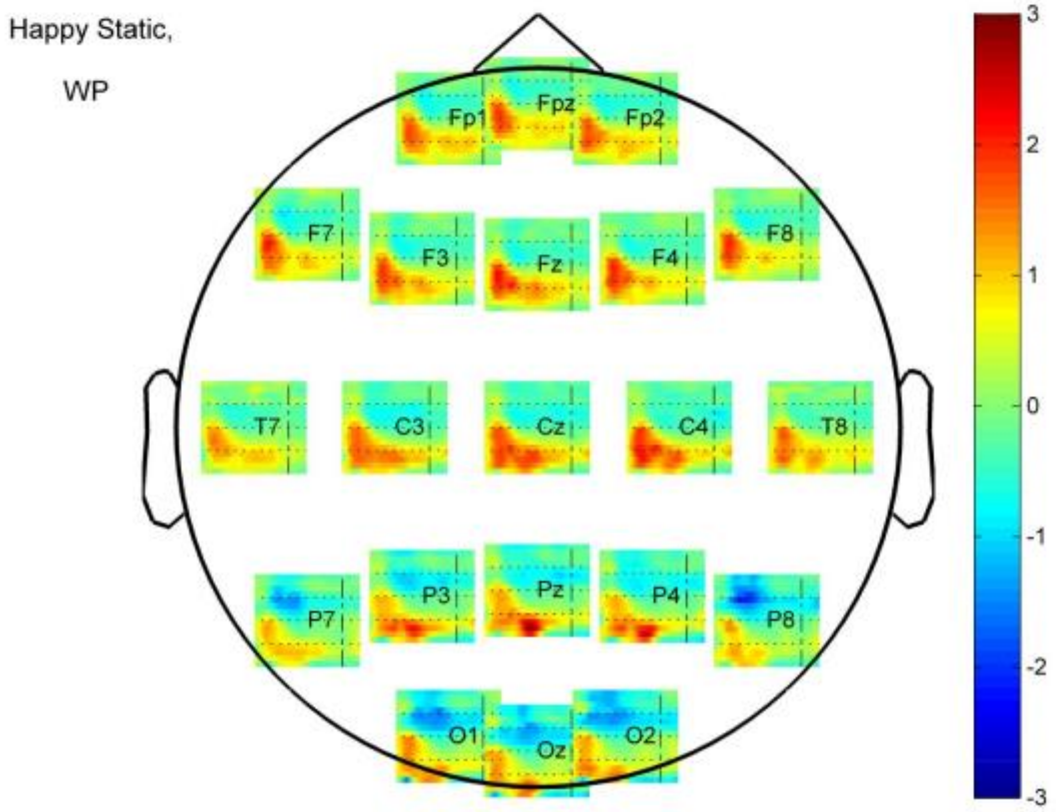

Figure G.

Topography with *PLI* (a) and *WP* (b) time-frequency diagrams (21 electrodes of the 10-20 international system) of the Angry Unnatural Dynamic condition.

a)

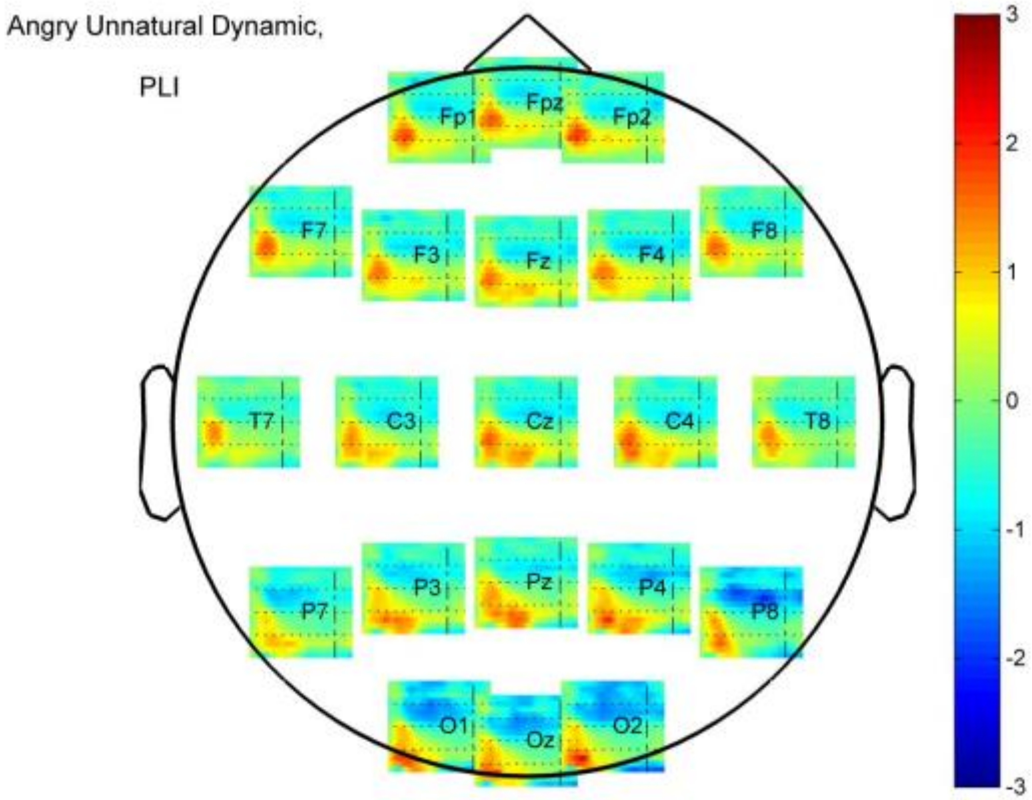


b)

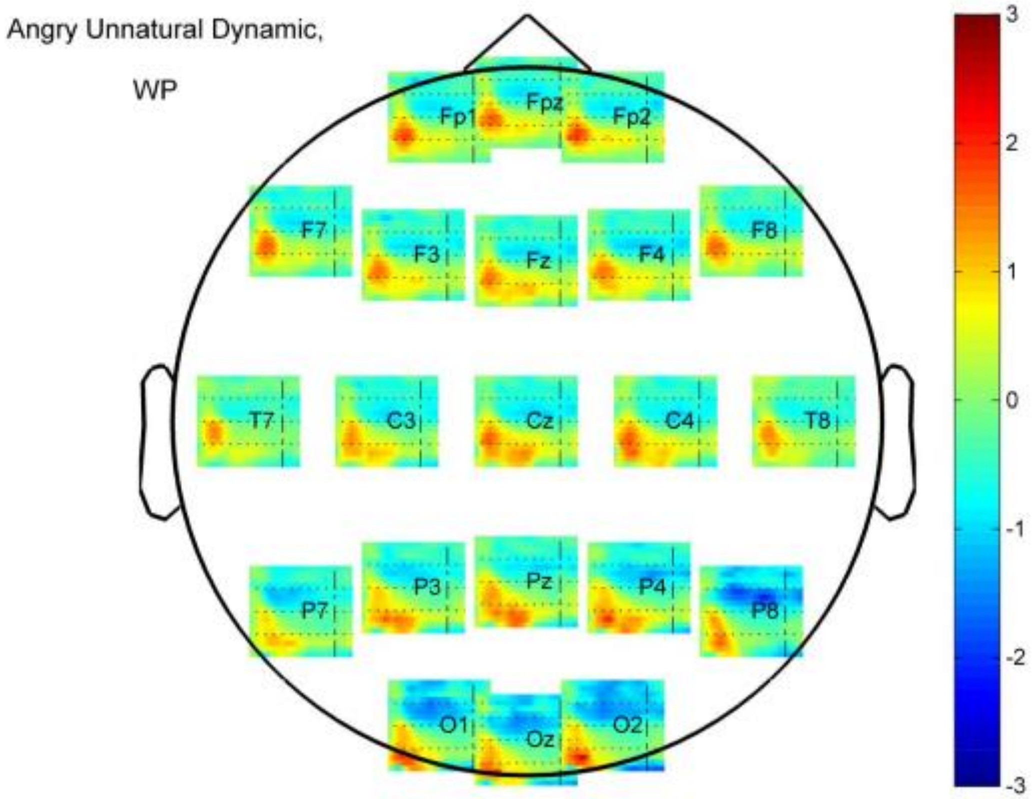

Figure H.

Topography with *PLI* (a) and *WP* (b) time-frequency diagrams (21 electrodes of the 10-20 international system) of the Happy Unnatural Dynamic condition.

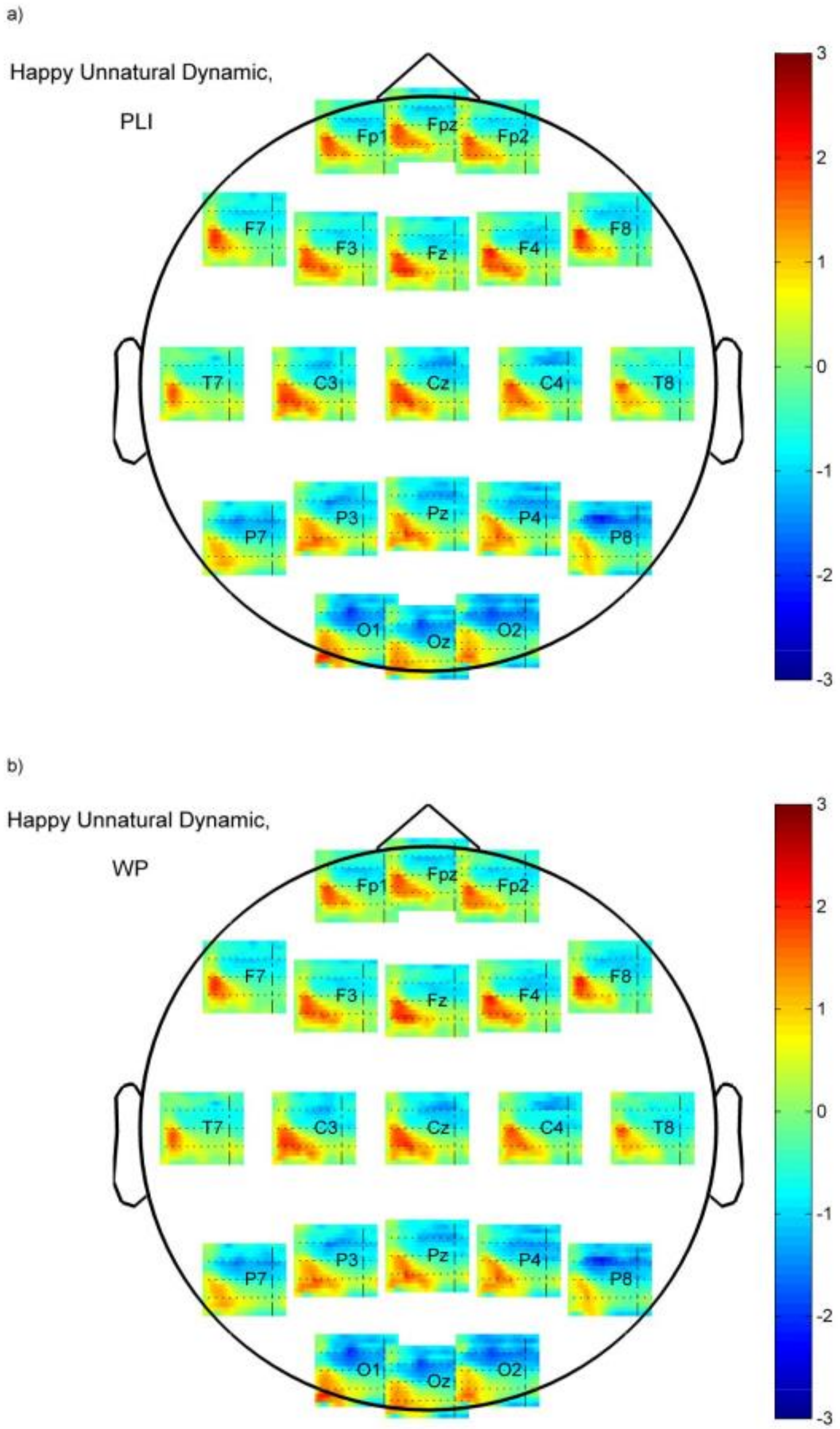

**Table A**

Validation rating for variable 'Angry'.

(Friedman test with "hsd" correction, using MATLAB function multcompare.m)

| 'Cond A' | 'Cond B' | 'lower 95% bound' | 'mean' | 'upper 95% bound' | 'p-value' |
|---|---|---|---|---|---|
| EXPRESSION | | | | | |
| 'angry' | 'happy' | 6,31841 | 9,83621 | 13,35401 | 0,00000* |
| MOTION | | | | | |
| 'dynamic' | 'static' | -2,90701 | 1,29957 | 5,50615 | 0,74922 |
| 'dynamic' | 'unnatural' | -6,99969 | -2,79310 | 1,41348 | 0,26487 |
| 'static' | 'unnatural' | -8,29926 | -4,09267 | 0,11391 | 0,05859 |
| EXPRESSION x MOTION | | | | | |
| 'angry-dynamic' | 'angry-static' | -3,73547 | 1,37931 | 6,49410 | 0,97279 |
| 'angry-dynamic' | 'angry-unnatural' | -5,53720 | -0,42241 | 4,69237 | 0,99990 |
| 'angry-dynamic' | 'happy-dynamic' | 6,35504 | 11,46983 | 16,58461 | 0,00000* |
| 'angry-dynamic' | 'happy-static' | 7,57487 | 12,68966 | 17,80444 | 0,00000* |
| 'angry-dynamic' | 'happy-unnatural' | 1,19125 | 6,30603 | 11,42082 | 0,00590 * |
| 'angry-static' | 'angry-unnatural' | -6,91651 | -1,80172 | 3,31306 | 0,91685 |
| 'angry-static' | 'happy-dynamic' | 4,97573 | 10,09052 | 15,20530 | 0,00000* |
| 'angry-static' | 'happy-static' | 6,19556 | 11,31034 | 16,42513 | 0,00000* |
| 'angry-static' | 'happy-unnatural' | -0,18806 | 4,92672 | 10,04151 | 0,06672 |
| 'angry-unnatural' | 'happy-dynamic' | 6,77746 | 11,89224 | 17,00703 | 0,00000* |
| 'angry-unnatural' | 'happy-static' | 7,99728 | 13,11207 | 18,22685 | 0,00000* |
| 'angry-unnatural' | 'happy-unnatural' | 1,61366 | 6,72845 | 11,84323 | 0,00245* |
| 'happy-dynamic' | 'happy-static' | -3,89496 | 1,21983 | 6,33461 | 0,98423 |
| 'happy-dynamic' | 'happy-unnatural' | -10,27858 | -5,16379 | -0,04901 | 0,04627* |
| 'happy-static' | 'happy-unnatural' | -11,49841 | -6,38362 | -1,26884 | 0,00505* |

**Table B**
Validation rating for variable 'Happy'.
(Friedman test with "hsd" correction, using MATLAB function multcompare.m)

| 'Cond A' | 'Cond B' | 'lower 95% bound' | 'mean' | 'upper 95% bound' | 'p-value' |
|---|---|---|---|---|---|
| EXPRESSION | | | | | |
| 'angry' | 'happy' | -14,14495 | -10,64080 | -7,13666 | 0,00000* |
| MOTION | | | | | |
| 'dynamic' | 'static' | -5,85404 | -1,66379 | 2,52646 | 0,62090 |
| 'dynamic' | 'unnatural' | -1,99628 | 2,19397 | 6,38421 | 0,43708 |
| 'static' | 'unnatural' | -0,33249 | 3,85776 | 8,04801 | 0,07862 |
| EXPRESSION x MOTION | | | | | |
| 'angry-dynamic' | 'angry-static' | -7,11216 | -2,01724 | 3,07768 | 0,86977 |
| 'angry-dynamic' | 'angry-unnatural' | -6,28027 | -1,18534 | 3,90958 | 0,98590 |
| 'angry-dynamic' | 'happy-dynamic' | -18,22423 | -13,12931 | -8,03439 | 0,00000* |
| 'angry-dynamic' | 'happy-static' | -19,53458 | -14,43966 | -9,34473 | 0,00000* |
| 'angry-dynamic' | 'happy-unnatural' | -12,65096 | -7,55603 | -2,46111 | 0,00034* |
| 'angry-static' | 'angry-unnatural' | -4,26303 | 0,83190 | 5,92682 | 0,99729 |
| 'angry-static' | 'happy-dynamic' | -16,20699 | -11,11207 | -6,01715 | 0,00000* |
| 'angry-static' | 'happy-static' | -17,51734 | -12,42241 | -7,32749 | 0,00000* |
| 'angry-static' | 'happy-unnatural' | -10,63372 | -5,53879 | -0,44387 | 0,02391* |
| 'angry-unnatural' | 'happy-dynamic' | -17,03889 | -11,94397 | -6,84904 | 0,00000* |
| 'angry-unnatural' | 'happy-static' | -18,34923 | -13,25431 | -8,15939 | 0,00000* |
| 'angry-unnatural' | 'happy-unnatural' | -11,46561 | -6,37069 | -1,27577 | 0,00493* |
| 'happy-dynamic' | 'happy-static' | -6,40527 | -1,31034 | 3,78458 | 0,97791 |
| 'happy-dynamic' | 'happy-unnatural' | 0,47835 | 5,57328 | 10,66820 | 0,02251* |
| 'happy-static' | 'happy-unnatural' | 1,78870 | 6,88362 | 11,97854 | 0,00165* |

**Table C**

Validation rating for variable 'Movement'.

(Friedman test with "hsd" correction, using MATLAB function multcompare.m)

| 'Cond A' | 'Cond B' | 'lower 95% bound' | 'mean' | 'upper 95% bound' | 'p-value' |
|---|---|---|---|---|---|
| EXPRESSION | | | | | |
| 'angry' | 'happy' | -6,31320 | -2,71839 | 0,87642 | 0,13831 |
| MOTION | | | | | |
| 'dynamic' | 'static' | -3,93444 | 0,36422 | 4,66289 | 0,97850 |
| 'dynamic' | 'unnatural' | -2,86548 | 1,43319 | 5,73186 | 0,71448 |
| 'static' | 'unnatural' | -3,22970 | 1,06897 | 5,36763 | 0,82933 |
| EXPRESSION x MOTION | | | | | |
| 'angry-dynamic' | 'angry-static' | -4,86037 | 0,36638 | 5,59313 | 0,99996 |
| 'angry-dynamic' | 'angry-unnatural' | -4,61899 | 0,60776 | 5,83451 | 0,99947 |
| 'angry-dynamic' | 'happy-dynamic' | -8,49399 | -3,26724 | 1,95951 | 0,47785 |
| 'angry-dynamic' | 'happy-static' | -8,13192 | -2,90517 | 2,32158 | 0,60922 |
| 'angry-dynamic' | 'happy-unnatural' | -6,23537 | -1,00862 | 4,21813 | 0,99404 |
| 'angry-static' | 'angry-unnatural' | -4,98537 | 0,24138 | 5,46813 | 0,99999 |
| 'angry-static' | 'happy-dynamic' | -8,86037 | -3,63362 | 1,59313 | 0,35315 |
| 'angry-static' | 'happy-static' | -8,49830 | -3,27155 | 1,95520 | 0,47631 |
| 'angry-static' | 'happy-unnatural' | -6,60175 | -1,37500 | 3,85175 | 0,97559 |
| 'angry-unnatural' | 'happy-dynamic' | -9,10175 | -3,87500 | 1,35175 | 0,28053 |
| 'angry-unnatural' | 'happy-static' | -8,73968 | -3,51293 | 1,71382 | 0,39260 |
| 'angry-unnatural' | 'happy-unnatural' | -6,84313 | -1,61638 | 3,61037 | 0,95109 |
| 'happy-dynamic' | 'happy-static' | -4,86468 | 0,36207 | 5,58882 | 0,99996 |
| 'happy-dynamic' | 'happy-unnatural' | -2,96813 | 2,25862 | 7,48537 | 0,82152 |
| 'happy-static' | 'happy-unnatural' | -3,33020 | 1,89655 | 7,12330 | 0,90658 |

**Table D**

Validation rating for variable ‘Naturalness’.

(Friedman test with “hsd” correction, using MATLAB function multcompare.m)

| 'Cond A' | 'Cond B' | 'lower 95% bound' | 'mean' | 'upper 95% bound' | 'p-value' |
|---|---|---|---|---|---|
| EXPRESSION | | | | | |
| 'angry' | 'happy' | -3,85282 | -0,25862 | 3,33558 | 0,88785 |
| MOTION | | | | | |
| 'dynamic' | 'static' | -4,84105 | -0,54310 | 3,75484 | 0,95280 |
| 'dynamic' | 'unnatural' | 3,85508 | 8,15302 | 12,45096 | 0,00000* |
| 'static' | 'unnatural' | 4,39818 | 8,69612 | 12,99406 | 0,00000* |
| EXPRESSION x MOTION | | | | | |
| 'angry-dynamic' | 'angry-static' | -1,14828 | 4,07759 | 9,30345 | 0,22678 |
| 'angry-dynamic' | 'angry-unnatural' | -7,35087 | -2,12500 | 3,10087 | 0,85635 |
| 'angry-dynamic' | 'happy-dynamic' | -9,16983 | -3,94397 | 1,28190 | 0,26132 |
| 'angry-dynamic' | 'happy-static' | 4,87758 | 10,10345 | 15,32932 | 0,00000* |
| 'angry-dynamic' | 'happy-unnatural' | -1,88104 | 3,34483 | 8,57069 | 0,45015 |
| 'angry-static' | 'angry-unnatural' | -8,08363 | -2,85776 | 2,36811 | 0,62616 |
| 'angry-static' | 'happy-dynamic' | -9,90259 | -4,67672 | 0,54914 | 0,10988 |
| 'angry-static' | 'happy-static' | 4,14482 | 9,37069 | 14,59656 | 0,00000* |
| 'angry-static' | 'happy-unnatural' | -11,42845 | -6,20259 | -0,97672 | 0,00938* |
| 'angry-unnatural' | 'happy-dynamic' | -13,24742 | -8,02155 | -2,79568 | 0,00018* |
| 'angry-unnatural' | 'happy-static' | 0,79999 | 6,02586 | 11,25173 | 0,01302* |
| 'angry-unnatural' | 'happy-unnatural' | -7,04483 | -1,81897 | 3,40690 | 0,92070 |
| 'happy-dynamic' | 'happy-static' | 7,00258 | 12,22845 | 17,45432 | 0,00000* |
| 'happy-dynamic' | 'happy-unnatural' | 8,82155 | 14,04741 | 19,27328 | 0,00000* |
| 'happy-static' | 'happy-unnatural' | -1,14828 | 4,07759 | 9,30345 | 0,22678 |

**Table E**
Error rates of standard stimuli.
(Friedman test with “hsd” correction, using MATLAB function multcompare.m)

| 'Cond A' | 'Cond B' | 'lower 95% bound' | 'mean' | 'upper 95% bound' | 'p-value' |
|---|---|---|---|---|---|
| EXPRESSION | | | | | |
| 'angry' | 'happy' | -4,51673 | -2,12644 | 0,26386 | 0,08123 |
| MOTION | | | | | |
| 'dynamic' | 'static' | -2,96392 | -0,10560 | 2,75271 | 0,99587 |
| 'dynamic' | 'unnatural' | -6,14064 | -3,28233 | -0,42401 | 0,01948* |
| 'static' | 'unnatural' | -6,03504 | -3,17672 | -0,31841 | 0,02493* |
| EXPRESSION x MOTION | | | | | |
| 'angry-dynamic' | 'angry-static' | -3,97112 | -0,49569 | 2,97974 | 0,99858 |
| 'angry-dynamic' | 'angry-unnatural' | -4,72974 | -1,25431 | 2,22112 | 0,90852 |
| 'angry-dynamic' | 'happy-dynamic' | -4,50991 | -1,03448 | 2,44094 | 0,95836 |
| 'angry-dynamic' | 'happy-static' | -4,22543 | -0,75000 | 2,72543 | 0,98999 |
| 'angry-dynamic' | 'happy-unnatural' | -9,82025 | -6,34483 | -2,86940 | 0,00000* |
| 'angry-static' | 'angry-unnatural' | -4,23405 | -0,75862 | 2,71680 | 0,98945 |
| 'angry-static' | 'happy-dynamic' | -4,01422 | -0,53879 | 2,93663 | 0,99788 |
| 'angry-static' | 'happy-static' | -3,72974 | -0,25431 | 3,22112 | 0,99995 |
| 'angry-static' | 'happy-unnatural' | -9,32456 | -5,84914 | -2,37371 | 0,00000* |
| 'angry-unnatural' | 'happy-dynamic' | -3,25560 | 0,21983 | 3,69525 | 0,99997 |
| 'angry-unnatural' | 'happy-static' | -2,97112 | 0,50431 | 3,97974 | 0,99846 |
| 'angry-unnatural' | 'happy-unnatural' | -8,56594 | -5,09052 | -1,61509 | 0,00043* |
| 'happy-dynamic' | 'happy-static' | -3,19094 | 0,28448 | 3,75991 | 0,99991 |
| 'happy-dynamic' | 'happy-unnatural' | -8,78577 | -5,31034 | -1,83492 | 0,00019* |
| 'happy-static' | 'happy-unnatural' | -9,07025 | -5,59483 | -2,11940 | 0,00000* |

**Table F**

Error rates of deviant stimuli.

(Friedman test with “hsd” correction, using MATLAB function multcompare.m)

| 'Cond A' | 'Cond B' | 'lower 95% bound' | 'mean' | 'upper 95% bound' | 'p-value' |
|---|---|---|---|---|---|
| EXPRESSION | | | | | |
| 'angry' | 'happy' | -3,20033 | 0,12644 | 3,45321 | 0,94062 |
| MOTION | | | | | |
| 'dynamic' | 'static' | -4,00832 | -0,03017 | 3,94797 | 0,99983 |
| 'dynamic' | 'unnatural' | -10,32297 | -6,34483 | -2,36668 | 0,00054* |
| 'static' | 'unnatural' | -10,29280 | -6,31466 | -2,33651 | 0,00058* |
| EXPRESSION x MOTION | | | | | |
| 'angry-dynamic' | 'angry-static' | -4,55255 | 0,28448 | 5,12151 | 0,99998 |
| 'angry-dynamic' | 'angry-unnatural' | -7,10858 | -2,27155 | 2,56548 | 0,76364 |
| 'angry-dynamic' | 'happy-dynamic' | -1,78530 | 3,05172 | 7,88875 | 0,46704 |
| 'angry-dynamic' | 'happy-static' | -2,13013 | 2,70690 | 7,54393 | 0,60203 |
| 'angry-dynamic' | 'happy-unnatural' | -12,20341 | -7,36638 | -2,52935 | 0,00021* |
| 'angry-static' | 'angry-unnatural' | -7,39306 | -2,55603 | 2,28099 | 0,66050 |
| 'angry-static' | 'happy-dynamic' | -2,06979 | 2,76724 | 7,60427 | 0,57832 |
| 'angry-static' | 'happy-static' | -2,41462 | 2,42241 | 7,25944 | 0,71046 |
| 'angry-static' | 'happy-unnatural' | -12,48789 | -7,65086 | -2,81383 | 0,00000* |
| 'angry-unnatural' | 'happy-dynamic' | 0,48625 | 5,32328 | 10,16030 | 0,02120* |
| 'angry-unnatural' | 'happy-static' | 0,14142 | 4,97845 | 9,81548 | 0,03936* |
| 'angry-unnatural' | 'happy-unnatural' | -9,93186 | -5,09483 | -0,25780 | 0,03212* |
| 'happy-dynamic' | 'happy-static' | -5,18186 | -0,34483 | 4,49220 | 0,99995 |
| 'happy-dynamic' | 'happy-unnatural' | -15,25513 | -10,41810 | -5,58107 | 0,00000* |
| 'happy-static' | 'happy-unnatural' | -14,91030 | -10,07328 | -5,23625 | 0,00000* |

**Table G**
SPSS output for a two-way repeated measures ANOVA on the logarithms of reaction times, with pairwise multiple comparisons of marginal means.

# General Linear Model

## Notes

| | | |
|---|---|---|
| Output Created | | 24-JUN-2017 21:10:39 |
| Comments | | |
| Input | Data | /Users/dionperd/Dropbox/Work/PostDocBerlin/Publications/BrainSynchronyFEEs/DataOnline/behavioral_data/reaction_time/reactionTimes.sav |
| | Active Dataset | DataSet1 |
| | Filter | <none> |
| | Weight | <none> |
| | Split File | <none> |
| | N of Rows in Working Data File | 29 |
| Missing Value Handling | Definition of Missing | User-defined missing values are treated as missing. |
| | Cases Used | Statistics are based on all cases with valid data for all variables in the model. |

| | | |
|---|---|---|
| Syntax | | GLM log_angry_Dynamic log_angry_Static log_angry_Unnatural log_happy_Dynamic log_happy_Static<br>log_happy_Unnatural<br>/WSFACTOR=EXPRESSION 2 Polynomial MOTION 3 Polynomial<br>/MEASURE=Reaction_Times<br>/METHOD=SSTYPE(3)<br>/EMMEANS=TABLES(OVERALL)<br>/EMMEANS=TABLES(EXPRESSION) COMPARE ADJ(BONFERRONI)<br>/EMMEANS=TABLES(MOTION) COMPARE ADJ(BONFERRONI)<br>/EMMEANS=TABLES(EXPRESSION*MOTION)<br>/CRITERIA=ALPHA(.05)<br>/WSDESIGN=EXPRESSION MOTION EXPRESSION*MOTION. |
| Resources | Processor Time | 00:00:00.02 |
| | Elapsed Time | 00:00:00.00 |

```
[DataSet1] /Users/dionperd/Dropbox/Work/PostDocBerlin/Publications/BrainSynchronyFEEs/DataOnline/behavioral_data/reaction_time/reactionTimes.sav
```

## Within-Subjects Factors

Measure: Reaction_Times

| EXPRESSION | MOTION | Dependent Variable |
|---|---|---|
| 1 | 1 | log_angry_Dynamic |
| | 2 | log_angry_Static |
| | 3 | log_angry_Unnatural |
| 2 | 1 | log_happy_Dynamic |
| | 2 | log_happy_Static |
| | 3 | log_happy_Unnatural |

## Multivariate Tests[a]

| Effect | | Value | F | Hypothesis df | Error df | Sig. |
|---|---|---|---|---|---|---|
| EXPRESSION | Pillai's Trace | .385 | 14.421[b] | 1.000 | 23.000 | .001 |
| | Wilks' Lambda | .615 | 14.421[b] | 1.000 | 23.000 | .001 |
| | Hotelling's Trace | .627 | 14.421[b] | 1.000 | 23.000 | .001 |
| | Roy's Largest Root | .627 | 14.421[b] | 1.000 | 23.000 | .001 |
| MOTION | Pillai's Trace | .881 | 81.817[b] | 2.000 | 22.000 | .000 |
| | Wilks' Lambda | .119 | 81.817[b] | 2.000 | 22.000 | .000 |
| | Hotelling's Trace | 7.438 | 81.817[b] | 2.000 | 22.000 | .000 |
| | Roy's Largest Root | 7.438 | 81.817[b] | 2.000 | 22.000 | .000 |
| EXPRESSION * MOTION | Pillai's Trace | .619 | 17.835[b] | 2.000 | 22.000 | .000 |
| | Wilks' Lambda | .381 | 17.835[b] | 2.000 | 22.000 | .000 |
| | Hotelling's Trace | 1.621 | 17.835[b] | 2.000 | 22.000 | .000 |
| | Roy's Largest Root | 1.621 | 17.835[b] | 2.000 | 22.000 | .000 |

a. Design: Intercept
 Within Subjects Design: EXPRESSION + MOTION + EXPRESSION * MOTION
b. Exact statistic

## Mauchly's Test of Sphericity[a]

Measure: Reaction_Times

| Within Subjects Effect | Mauchly's W | Approx. Chi-Square | df | Sig. | Epsilon[b] | |
|---|---|---|---|---|---|---|
| | | | | | Greenhouse-Geisser | Huynh-Feldt |

| | | | | | | |
|---|---|---|---|---|---|---|
| EXPRESSION | 1.000 | .000 | 0 | . | 1.000 | 1.000 |
| MOTION | .709 | 7.564 | 2 | .023 | .775 | .820 |
| EXPRESSION * MOTION | .236 | 31.755 | 2 | .000 | .567 | .577 |

## Mauchly's Test of Sphericity[a]

Measure: Reaction_Times

| Within Subjects Effect | Epsilon Lower-bound |
|---|---|
| EXPRESSION | 1.000 |
| MOTION | .500 |
| EXPRESSION * MOTION | .500 |

Tests the null hypothesis that the error covariance matrix of the orthonormalized transformed dependent variables is proportional to an identity matrix.[a]

a. Design: Intercept

Within Subjects Design: EXPRESSION + MOTION + EXPRESSION * MOTION

b. May be used to adjust the degrees of freedom for the averaged tests of significance. Corrected tests are displayed in the Tests of Within-Subjects Effects table.

## Tests of Within-Subjects Effects

Measure: Reaction_Times

| Source | | Type III Sum of Squares | df | Mean Square | F | Sig. |
|---|---|---|---|---|---|---|
| EXPRESSION | Sphericity Assumed | .143 | 1 | .143 | 14.421 | .001 |
| | Greenhouse-Geisser | .143 | 1.000 | .143 | 14.421 | .001 |
| | Huynh-Feldt | .143 | 1.000 | .143 | 14.421 | .001 |
| | Lower-bound | .143 | 1.000 | .143 | 14.421 | .001 |
| Error(EXPRESSION) | Sphericity Assumed | .228 | 23 | .010 | | |
| | Greenhouse-Geisser | .228 | 23.000 | .010 | | |
| | Huynh-Feldt | .228 | 23.000 | .010 | | |
| | Lower-bound | .228 | 23.000 | .010 | | |
| MOTION | Sphericity Assumed | 2.406 | 2 | 1.203 | 122.043 | .000 |
| | Greenhouse-Geisser | 2.406 | 1.549 | 1.553 | 122.043 | .000 |
| | Huynh-Feldt | 2.406 | 1.640 | 1.467 | 122.043 | .000 |
| | Lower-bound | 2.406 | 1.000 | 2.406 | 122.043 | .000 |
| Error(MOTION) | Sphericity Assumed | .454 | 46 | .010 | | |
| | Greenhouse-Geisser | .454 | 35.633 | .013 | | |

| | | | | | | |
|---|---|---|---|---|---|---|
| | Huynh-Feldt | .454 | 37.723 | .012 | | |
| | Lower-bound | .454 | 23.000 | .020 | | |
| EXPRESSION * MOTION | Sphericity Assumed | .258 | 2 | .129 | 18.827 | .000 |
| | Greenhouse-Geisser | .258 | 1.134 | .228 | 18.827 | .000 |
| | Huynh-Feldt | .258 | 1.153 | .224 | 18.827 | .000 |
| | Lower-bound | .258 | 1.000 | .258 | 18.827 | .000 |
| Error(EXPRESSION* MOTION) | Sphericity Assumed | .316 | 46 | .007 | | |
| | Greenhouse-Geisser | .316 | 26.079 | .012 | | |
| | Huynh-Feldt | .316 | 26.520 | .012 | | |
| | Lower-bound | .316 | 23.000 | .014 | | |

## Tests of Within-Subjects Contrasts

Measure: Reaction_Times

| Source | EXPRESSION | MOTION | Type III Sum of Squares | df | Mean Square | F | Sig. |
|---|---|---|---|---|---|---|---|
| EXPRESSION | Linear | | .143 | 1 | .143 | 14.421 | .001 |
| Error(EXPRESSION) | Linear | | .228 | 23 | .010 | | |
| MOTION | | Linear | .335 | 1 | .335 | 44.059 | .000 |
| | | Quadratic | 2.071 | 1 | 2.071 | 171.065 | .000 |
| Error(MOTION) | | Linear | .175 | 23 | .008 | | |
| | | Quadratic | .278 | 23 | .012 | | |
| EXPRESSION * MOTION | Linear | Linear | .256 | 1 | .256 | 22.345 | .000 |
| | | Quadratic | .003 | 1 | .003 | 1.105 | .304 |
| Error(EXPRESSION* MOTION) | Linear | Linear | .263 | 23 | .011 | | |
| | | Quadratic | .052 | 23 | .002 | | |

## Tests of Between-Subjects Effects

Measure: Reaction_Times
Transformed Variable: Average

| Source | Type III Sum of Squares | df | Mean Square | F | Sig. |
|---|---|---|---|---|---|
| Intercept | .007 | 1 | .007 | .075 | .786 |
| Error | 2.115 | 23 | .092 | | |

# Estimated Marginal Means

## 1. Grand Mean

Measure: Reaction_Times

| Mean | Std. Error | 95% Confidence Interval Lower Bound | 95% Confidence Interval Upper Bound |
|---|---|---|---|
| .007 | .025 | -.045 | .059 |

# 2. EXPRESSION

## Estimates

Measure: Reaction_Times

| EXPRESSION | Mean | Std. Error | 95% Confidence Interval Lower Bound | 95% Confidence Interval Upper Bound |
|---|---|---|---|---|
| 1 | .038 | .024 | -.012 | .089 |
| 2 | -.025 | .029 | -.084 | .034 |

## Pairwise Comparisons

Measure: Reaction_Times

| (I) EXPRESSION | (J) EXPRESSION | Mean Difference (I-J) | Std. Error | Sig.[b] | 95% Confidence Interval for Difference[b] Lower Bound | 95% Confidence Interval for Difference[b] Upper Bound |
|---|---|---|---|---|---|---|
| 1 | 2 | .063* | .017 | .001 | .029 | .097 |
| 2 | 1 | -.063* | .017 | .001 | -.097 | -.029 |

Based on estimated marginal means

*. The mean difference is significant at the

b. Adjustment for multiple comparisons: Bonferroni.

## Multivariate Tests

| | Value | F | Hypothesis df | Error df | Sig. |
|---|---|---|---|---|---|
| Pillai's trace | .385 | 14.421[a] | 1.000 | 23.000 | .001 |
| Wilks' lambda | .615 | 14.421[a] | 1.000 | 23.000 | .001 |
| Hotelling's trace | .627 | 14.421[a] | 1.000 | 23.000 | .001 |
| Roy's largest root | .627 | 14.421[a] | 1.000 | 23.000 | .001 |

Each F tests the multivariate effect of EXPRESSION. These tests are based on the linearly independent pairwise comparisons among the estimated marginal means.
a. Exact statistic

# 3. MOTION

## Estimates

Measure: Reaction_Times

| MOTION | Mean | Std. Error | 95% Confidence Interval | |
|---|---|---|---|---|
| | | | Lower Bound | Upper Bound |
| 1 | .033 | .028 | -.026 | .091 |
| 2 | -.163 | .020 | -.204 | -.122 |
| 3 | .151 | .034 | .081 | .221 |

## Pairwise Comparisons

Measure: Reaction_Times

| (I) MOTION | (J) MOTION | Mean Difference (I-J) | Std. Error | Sig.[b] | 95% Confidence Interval for Difference[b] | |
|---|---|---|---|---|---|---|
| | | | | | Lower Bound | Upper Bound |
| 1 | 2 | .195* | .017 | .000 | .152 | .239 |
| | 3 | -.118* | .018 | .000 | -.164 | -.072 |
| 2 | 1 | -.195* | .017 | .000 | -.239 | -.152 |
| | 3 | -.314* | .025 | .000 | -.378 | -.249 |
| 3 | 1 | .118* | .018 | .000 | .072 | .164 |
| | 2 | .314* | .025 | .000 | .249 | .378 |

Based on estimated marginal means
*. The mean difference is significant at the
b. Adjustment for multiple comparisons: Bonferroni.

## Multivariate Tests

| | Value | F | Hypothesis df | Error df | Sig. |
|---|---|---|---|---|---|
| Pillai's trace | .881 | 81.817[a] | 2.000 | 22.000 | .000 |
| Wilks' lambda | .119 | 81.817[a] | 2.000 | 22.000 | .000 |
| Hotelling's trace | 7.438 | 81.817[a] | 2.000 | 22.000 | .000 |
| Roy's largest root | 7.438 | 81.817[a] | 2.000 | 22.000 | .000 |

Each F tests the multivariate effect of MOTION. These tests are based on the linearly independent pairwise comparisons among the estimated marginal means.

a. Exact statistic

## 4. EXPRESSION * MOTION

Measure: Reaction_Times

| EXPRESSION | MOTION | Mean | Std. Error | 95% Confidence Interval Lower Bound | 95% Confidence Interval Upper Bound |
|---|---|---|---|---|---|
| 1 | 1 | .113 | .030 | .052 | .174 |
| | 2 | -.125 | .020 | -.166 | -.084 |
| | 3 | .128 | .030 | .066 | .190 |
| 2 | 1 | -.048 | .028 | -.106 | .011 |
| | 2 | -.200 | .022 | -.246 | -.154 |
| | 3 | .174 | .047 | .077 | .270 |
